# Integrated transcriptomic, untargeted and targeted metabolomic analyses reveal seasonal regulatory mechanisms of vascular cambium activity in woody plants: insights from *Schima superba*

**DOI:** 10.3389/fpls.2025.1727826

**Published:** 2026-01-22

**Authors:** Yuanhang Wu, Qingyu Yan, Jingjing Zou, Hongguo Chen, Yingting Zhang, Hui Xia

**Affiliations:** 1 College of Nuclear Technology & Chemistry and Biology, Hubei University of Science and Technology, Xianning, China; 2 State Key Laboratory of Woody Oil Resources Utilisation, College of Forestry, Central South University of Forestry and Technology, Changsha, China

**Keywords:** metabolome, transcriptome, phenylpropanoid biosynthesis, plant hormone signal transduction, seasonal cambial activity

## Abstract

**Introduction:**

The vascular cambium is a pivotal tissue that drives secondary growth in woody plants, directly determining wood structure and properties. *Schima superba*, a valuable timber species in China, holds considerable ecological and economic importance. However, the seasonal activity of its cambium remains poorly understood.

**Methods:**

To elucidate the underlying molecular and metabolic regulatory mechanisms, we integrated untargeted metabolomics, lignin-targeted metabolomics, and transcriptomic analyses.

**Results:**

Untargeted metabolomic profiling identified 2,178 differentially synthesised metabolites (DSMs), predominantly associated with amino acid metabolism, fatty acid metabolism, phenylpropanoid biosynthesis (PB), and plant hormone signal transduction (PHST). Lignin-targeted metabolomics detected 8 DSMs, most of which initially increased and subsequently declined, peaking in August, with the exception of sinapyl alcohol and L-phenylalanine. Transcriptomic analysis identified 19,609 differentially expressed genes (DEGs), primarily enriched in PB and PHST pathways. Key PB-related structural genes, including *SsPAL*, *SsC4H*, *Ss4CL*, *SsCOMT*, and *SsCAD*, exhibited peak expression during the cambial active period, correlating with lignin intermediate accumulation and indicating tight coordination between transcriptional activity and metabolic flux during lignification. PHST-related genes involved in auxin (AUX), cytokinin (CTK), and gibberellin (GA) signalling, such as *SsARF*, *SsSAUR*, and *SsARR-B*, displayed stage-specific expression patterns regulating cambial cell expansion and division. Weighted gene coexpression network analysis (WGCNA) further identified core transcription factors, including *SsMYB*, *SsbHLH*, *SsWOX*, *SsGRAS*, *SsSAUR*, and *SsNF-YB*, as regulators of seasonal cambial activity.

**Discussion:**

These findings provide a comprehensive framework for understanding seasonal cambial activity in *S. superba*, and offer valuable insights into the regulatory mechanisms of vascular cambium activity in woody plants, thereby supporting targeted wood quality improvement and advancing our understanding of plant developmental biology.

## 1 Introduction

Throughout their development, trees continuously increase in height and stem diameter, with diameter growth largely driven by the activity of the vascular cambium. This secondary growth represents a fundamental process in woody plants. As the central tissue responsible for wood formation, the vascular cambium has attracted considerable attention in recent decades (Aref et al., 2014; Fischer et al., 2019; Wang, 2020, Wang et al., 2021b). Situated between the xylem and phloem, it comprises a layer of undifferentiated, actively dividing cells that generate secondary xylem inwardly and secondary phloem outwardly (Fischer et al., 2019; Mizrachi and Myburg, 2016). Rather than referring to cambium initiation or establishment, cambial activity reflects the dynamic balance between cell division and differentiation, which varies markedly across seasons in perennial woody plants. These seasonal fluctuations in cambial activity not only influence wood structure and mechanical properties but also play critical roles in water and nutrient transport, mechanical support, and plant responses to environmental stresses (Ding et al., 2024; Qaderi et al., 2019). Therefore, elucidating the molecular regulatory mechanisms underlying seasonal vascular cambium activity and its regulation is essential for a comprehensive understanding of woody plant growth and provides a foundation for forest genetic improvement and sustainable forestry management.

The initiation, maintenance, and cessation of vascular cambium activity are critical determinants of wood quantity and quality. In high-latitude regions, cambial development exhibits pronounced seasonal rhythms and stage-specific characteristics (Alvarado et al., 2025; Guo et al., 2022; Yang et al., 2024a; Zhang et al., 2022), regulated by complex, multi-level mechanisms encompassing cellular, physiological, biochemical, and molecular processes (Chen et al., 2021; Kayal and El-Settawy, 2017). In temperate and subtropical trees, the annual cambial cycle is typically divided into five recognisable stages: dormancy, reactivation, active division, secondary wall thickening and differentiation, and pre-dormancy (Begum et al., 2007; Plomion et al., 2001; Rossi et al., 2003). These stages differ markedly in cell division rate, differentiation potential, lignification, hormonal responsiveness, and cell wall biosynthetic activity, accompanied by dynamic transcriptional programmes and metabolic shifts (Alvarado et al., 2025; Plomion et al., 2001). Much of the research has focused on model species such as *Arabidopsis thaliana* (Mao et al., 2019; Suer et al., 2011), and economically important trees including poplar (*Populus*) (Begum et al., 2007; Chen et al., 2021; Hu et al., 2022), eucalypt (*Eucalyptus*) (Foucart et al., 2006), Chinese fir (*Cunninghamia lanceolata*) (Qiu et al., 2013; Yang et al., 2024a, b, 2025) and Chinese cedar (*Cryptomeria fortunei*) (Yang et al., 2024c; Zhang et al., 2022). Phytohormones including auxin (AUX), cytokinin (CTK), gibberellin (GA), and ethylene (ETH), play central roles in stem cell maintenance, division, and vascular tissue differentiation (Miyashima et al., 2013; Mizrachi and Myburg, 2016; Wang et al., 2021a; Zhang et al., 2024a, b). Furthermore, transcription factor (TF) families such as class III homeodomain-leucine zipper (*HD-ZIP III*) (Guo et al., 2022; Miyashima et al., 2019; Robischon et al., 2011), WUSCHEL-related homeobox (*WOX*) (Hu et al., 2021; Petzold et al., 2018; Suer et al., 2011), myeloblastosis (*MYB*) (Yang et al., 2025; Zhang et al., 2022, 2024b), and NAM, ATAF1/2, and CUC2 (*NAC*) (Guo et al., 2022; Kubo et al., 2005; Yang et al., 2023) are pivotal for cell fate determination and xylem/phloem differentiation. Stage-specific activation of secondary metabolic pathways, particularly phenylpropanoid metabolism linked to lignin biosynthesis (Emiliani et al., 2011; Zhang et al., 2024a), further elucidates the metabolic regulation underlying wood formation.

With the rapid advancement of multi-omics technologies, integrating multi-layered datasets has become a vital strategy for elucidating complex plant developmental processes (Li et al., 2024; Wu et al., 2024). RNA sequencing (RNA-seq), a high-throughput expression profiling approach, enables comprehensive characterisation of gene expression dynamics across temporal and spatial scales. This technique reveals signalling pathways and key regulatory factors controlling vascular cambium activity, providing a powerful tool for investigating the molecular mechanisms underlying woody plant growth (Qiu et al., 2013; Schrader et al., 2004). For instance, Chano et al. (2017) employed transcriptome analysis to uncover the molecular basis of traumatic wood formation in conifers, while studies in poplar have elucidated transcriptional regulation during the transition from active cambial growth to dormancy (Druart et al., 2007; Ruttink et al., 2007). Expression sequence tag (EST)-based analyses have further expanded understanding of gene regulatory networks in wood formation (Foucart et al., 2006; Kirst et al., 2003), gradually identifying key genes associated with cambial development (Schrader et al., 2004). At the metabolic level, untargeted metabolomics enables the identification of stage-specific metabolic reprogramming and facilitates the discovery of functional metabolites, whereas targeted metabolomics provides precise quantification of lignin-related compounds, such as pinoresinol, coniferaldehyde, and *p*-coumaryl alcohol, whose abundance reflects the metabolic foundation of wood formation (Ma et al., 2023; Xu et al., 2023). Integration of transcriptomic and metabolomic datasets enables the construction of multi-layered regulatory networks, elucidating the coordination between gene expression and metabolite accumulation, and providing systematic insights into wood formation (Ma et al., 2023; Xiao et al., 2021; Xu et al., 2023; Zhang et al., 2024a, b). Nevertheless, the regulatory mechanisms underlying the seasonal activity of the vascular cambium in native Chinese broadleaf species remains poorly characterised, with most existing networks derived from model or economically important trees, limiting their applicability for precise molecular improvement of local species.

*Schima superba*, a member of the Theaceae family, is an evergreen broadleaf tree widely distributed in subtropical China. It is characterised by rapid growth, dense and resilient wood, strong ecological adaptability, and resistance to fire and insect damage, making it valuable for ecological restoration, timber production, and urban greening in southern China (Bai et al., 2024; Wang et al., 2023). In recent years, *S. superba* has been incorporated into sustainable forestry systems and local species breeding programmes, establishing itself as a typical “dual-purpose ecological and economic” species. Previous studies indicate that it enters the mature wood formation stage after approximately 16 years (Wang et al., 2021). Transcriptome analyses have identified candidate genes related to phenylpropanoid and cellulose biosynthesis (e.g., *Ss4CL2* (4-coumarate CoA ligase), *SsCSL1* (cellulose synthase-like), and *SsCSL2*) and several potential regulatory factors (e.g., *SsDELLA2*, *SsDELLA3*, *SsDELLA5*, and *SsNAM1*) in individuals with differing wood accumulation (Bai et al., 2024). Nevertheless, research on the seasonal activity of the vascular cambium in *S. superba* remains limited, thereby constraining its genetic improvement and targeted wood production.

In this study, we systematically investigated five representative seasonal activity stages of the vascular cambium in *S. superba* using integrated transcriptomic and metabolomic analyses. Our objectives were: (1) to characterise stage-specific gene expression dynamics through high-throughput RNA-seq, construct a temporal transcriptome atlas, and identify key regulatory factors and functional modules using WGCNA; (2) to integrate lignin-targeted metabolomics with untargeted metabolomics, analyse stage-specific differential metabolites, and correlate them with gene expression; and (3) to construct regulatory networks for the five stages, elucidating gene-driven metabolic regulation during secondary growth and identifying potential biomarkers. This study provides a theoretical framework for understanding the genetic regulation of wood formation in *S. superba*, expands knowledge of vascular cambium biology in broadleaf species, and offers valuable insights for genetic improvement and the targeted cultivation of high-quality timber.

## 2 Materials and methods

### 2.1 Experimental materials

**Figure 1:**
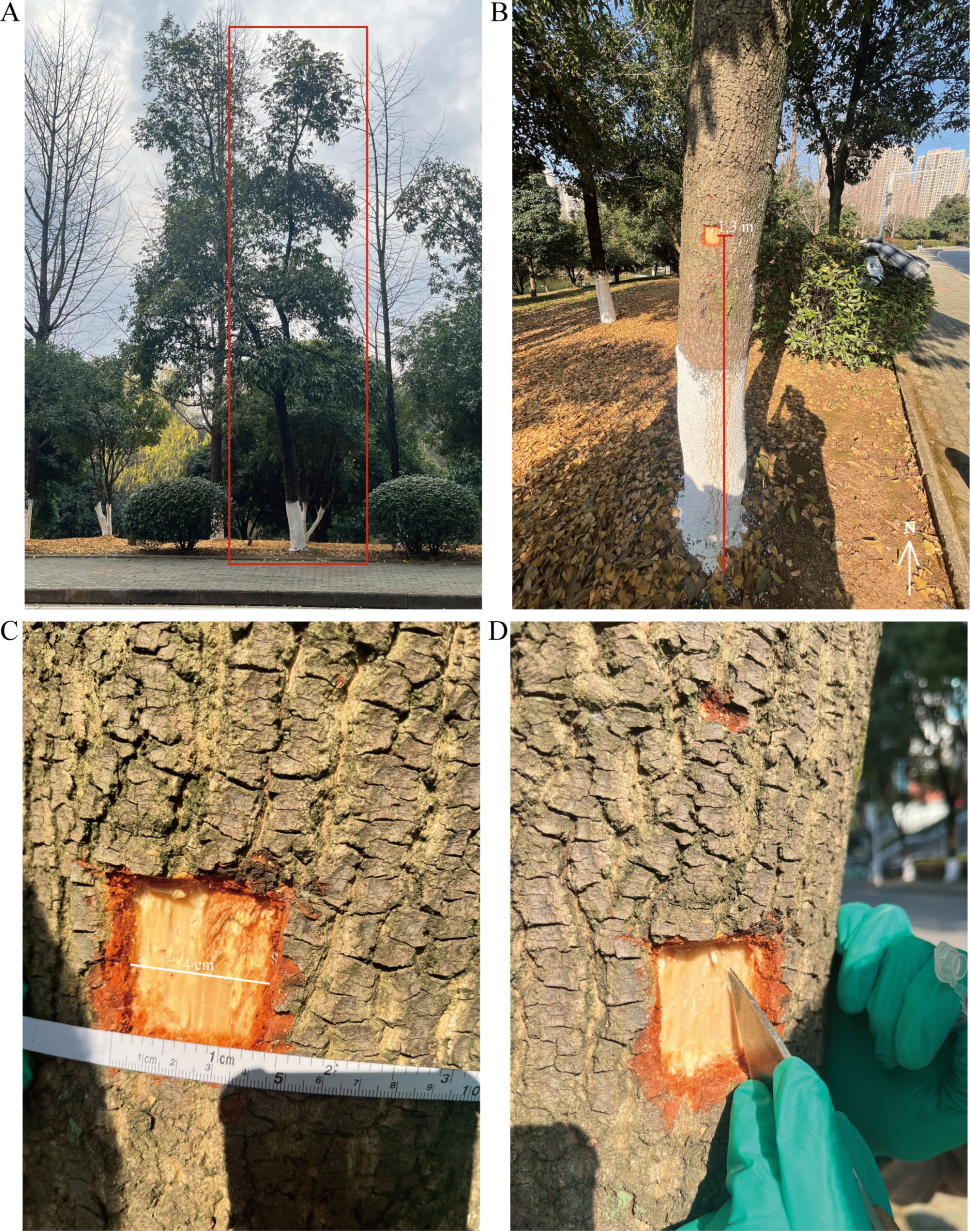
Sampling of vascular cambium in *S. superba*. **(A)** Whole-plant photograph of the sampled *S. superba* individual; **(B)** Sampling height on the trunk; **(C, D)** Photographs of the collected cambial tissue samples.

Twenty-year-old *S. superba* trees growing on the campus of Hubei University of Science and Technology (114°20′28.98″E, 29°50′59.88″N, Xianning, Hubei, China) were selected as experimental material (**Figure 1A**). Cambial samples were collected at five key seasonal activity stages (15 April, 15 June, 15 August, 15 October, and 15 December) between 09:30 and 10:00 (Guo et al., 2022; Shi et al., 2021; Xi and Zhao, 2012; Zeng et al., 2025; Zhong et al., 2011). At each time point, three independent biological replicates were collected from the south-facing trunk at a height of 1.2–1.4 m between 10:00 and 10:30 am (**Figure 1B**). The bark and phloem were gently removed using a sterile scalpel to expose the vascular cambium (**Figure 1C**). The cambial tissue, appearing as a thin, translucent layer between the phloem and xylem, was carefully scraped 3–5 times, with only the tissue obtained during the third to fifth scrapes retained to minimise contamination from adjacent tissues (**Figure 1D**). These samples were immediately flash-frozen in liquid nitrogen and stored at −80°C for metabolomic and transcriptomic analyses. At each stage, three biological replicates were collected from the same individual tree to ensure data reliability and representativeness.

### 2.2 Sample preparation and extraction

Samples were vacuum freeze-dried using a lyophilizer (Scientz-100F) with a tissue grinder (MM 400, Retsch) at 30 Hz for 1.5 minutes. A 50 mg portion of the powder was extracted with 1,200 μL of −20°C pre-chilled 70% methanol containing internal standards, with the volume adjusted proportionally for smaller samples. The mixture was vortexed for 30 seconds every 30 minutes over six cycles. Following extraction, samples were centrifuged at 12,000 rpm for 3 minutes, and the supernatant was collected, filtered through a 0.22 μm microporous membrane, and transferred to autosampler vials for ultra-performance liquid chromatography coupled with tandem mass spectrometry (UPLC–MS/MS) analysis.

### 2.3 Untargeted metabolomics detection

All samples were analysed using an LC-MS system. Chromatographic separation was performed on a Waters ACQUITY UPLC HSS T3 column (1.8 µm, 2.1 mm × 100 mm) maintained at 40°C, with a flow rate of 0.40 mL min^–1^ and an injection volume of 4 µL. A binary solvent system was employed for gradient elution, with mobile phase A comprising ultrapure water containing 0.1% formic acid, and mobile phase B comprising acetonitrile containing 0.1% formic acid. The 10-min gradient program was as follows: 0.0 min, 95% A; 5.0 min, 35% A; 6.0 min, 0% A; 7.5 min, 0% A; and 7.6 min, 95% A.

Data acquisition was performed in information-dependent acquisition mode using Analyst TF 1.7.1 software (Sciex, Concord, ON, Canada). MS parameters were set as follows: ion source gases 1 and 2 at 50 and 60 psi, curtain gas at 35 psi, source temperature at 550°C, and declustering potential at 80 V. The ion spray voltage was 5,500 V in positive mode and −4,500 V in negative mode. TOF MS scans were acquired over a mass range of 50–1,250 Da with an accumulation time of 200 ms and dynamic background subtraction enabled. Product ion scans were performed over the same mass range with an accumulation time of 40 ms, collision energy of 30 V (spread 15 V), unit resolution, an intensity threshold of 100 cps, isotope exclusion within 4 Da, a mass tolerance of 50 mDa, and monitoring of a maximum of 12 candidate ions per cycle.

### 2.4 Untargeted metabolomic analysis

All data were scaled to unit variance and subjected to unsupervised principal component analysis (PCA) using the prcomp function in R (www.r-project.org). Hierarchical clustering analysis (HCA) of both samples and metabolites was visualised as heatmaps with dendrograms. Pearson correlation coefficients (PCCs) between samples were calculated using the cor function in R and similarly displayed as heatmaps. Both HCA and PCC visualisations were generated using the ComplexHeatmap R package. For HCA, normalised metabolite signal intensities (unit variance-scaled) were presented in heatmap format.

Differentially synthesised metabolites (DSMs) between groups were identified based on variable importance in projection (VIP) scores > 1 and |Log_2_ fold change| ≥ 1.0. Orthogonal partial least squares discriminant analysis (OPLS-DA) and partial least squares discriminant analysis (PLS-DA) were performed using the MetaboAnalystR package in R, with log_2_ transformation and mean-centering applied prior to analysis. OPLS-DA results included score plots and permutation plots, from which VIP values were extracted. To prevent overfitting, a permutation test with 200 iterations was performed. Identified metabolites were annotated using the KEGG compound database (http://www.kegg.jp/kegg/compound/) and mapped to the KEGG pathway database (http://www.kegg.jp/kegg/pathway.html).

### 2.5 Lignin-targeted metabolomics assays

Lignin-targeted metabolites were analysed using UPLC-MS/MS (ExionLC™ AD, https://sciex.com.cn/). Chromatographic separation was performed on an Agilent SB-C18 column (1.8 µm, 2.1 mm × 100 mm). The mobile phase comprised solvent A (ultrapure water with 0.1% formic acid) and solvent B (acetonitrile with 0.1% formic acid). The gradient program was as follows: 0.0 min, 5% B; increased linearly to 95% B over 9.0 min and held at 95% B for 1 min; 10.0–11.1 min, decreased to 5% B and equilibrated at 5% B until 14.0 min. The flow rate was 0.35 mL min^–1^, the column temperature was maintained at 40°C, and the injection volume was 2 μL. The eluent was directly introduced into an ESI triple quadrupole linear ion trap (QTRAP) mass spectrometer.

MS analysis was performed on an API 6,500 QTRAP UPLC/MS/MS system equipped with an ESI Turbo Ion-Spray interface, operating in both positive and negative ion modes. The ESI source parameters were set as follows: ion source, turbo spray; source temperature, 550°C; ion spray voltage, 5,500 V; gas I, gas II, and curtain gas at 50, 60, and 30 psi, respectively; collision-activated dissociation, high. The instrument was tuned and mass-calibrated using 10 and 100 μM polypropylene glycol solutions in both triple quadrupole (QQQ) and linear ion trap (LIT) modes. Quantification was performed in multiple reaction monitoring (MRM) mode on the QQQ, with nitrogen as the collision gas at 5 psi.

### 2.6 Lignin-targeted metabolomics analysis

Raw MS data were processed using Analyst 1.6.3 software. Metabolite identification and quantification were performed based on an in-house database (MWDB, Metware Database). Peak areas for all metabolites were integrated, and signals of the same metabolite across different samples were corrected to ensure consistency (Fraga et al., 2010). Declustering potential and collision energy were further optimised. For each stage, specific MRM channels were monitored according to the eluted metabolites.

Quality control (QC) samples were prepared by pooling aliquots of all sample extracts to evaluate reproducibility under identical experimental conditions. During MS analysis, a QC sample was injected after every ten experimental samples to monitor analytical repeatability. Fold changes were calculated for each metabolite, and statistical significance was evaluated using either the Wilcoxon rank-sum test or Student’s t-test. Metabolites with fold change ≥ 2 or ≤ 0.5 were considered DSMs.

### 2.7 RNA extraction and transcriptome sequencing

Total RNA was extracted using the Qiagen RNeasy Plant Mini Kit (Qiagen, Hilden, Germany), with genomic DNA removed during extraction using the RNase-free DNase Kit (Qiagen). RNA quality was initially assessed by 1% agarose gel electrophoresis, ensuring the presence of clear 28S and 18S rRNA bands. RNA integrity and concentration were further evaluated using an Agilent 2100 Bioanalyzer. Qualified RNA samples were transported on dry ice to igenebook Co. (Wuhan, Hubei, China) for cDNA library construction and high-throughput sequencing on the Illumina NovaSeq platform.

### 2.8 Transcriptomic analysis

Raw image data generated from high-throughput sequencing were converted into raw sequencing reads via base calling. Adapter sequences and low-quality reads were removed using Cutadapt *v*1.11 (Martin, 2011) with the following parameters: -a R1_adapter -A R2_adapter -m 20 –max-n 0.05 -q 20. QC of the filtered reads was performed using FastQC *v*0.11.5 (Andrews, 2010), and summary statistics were generated for both raw and high-quality clean reads. For *de novo* transcriptomic analysis in the absence of a reference genome, high-quality RNA-seq reads were assembled using Trinity *v*2.4.0 (http://trinityrnaseq.sourceforge.net/) (Grabherr et al., 2011) to generate contigs and singletons, and the corresponding protein sequences were predicted. The longest transcript for each gene was selected as the representative unigene. Clean reads were then aligned to the assembled Trinity.fasta using Bowtie2 *v*2.2.9 (Langmead and Salzberg, 2012). Protein sequences were annotated against public databases using DIAMOND (Buchfink et al., 2015) with an e-value threshold of < 10^-5^, retrieving functional information from the non-redundant (NR), gene ontology (GO), and Kyoto encyclopedia of genes and genomes (KEGG) databases. TFs were predicted using PlantTFDB *v*5.0 (https://planttfdb.gao-lab.org/prediction.php) (Tian et al., 2020). Transcript abundance for each gene was calculated as fragments per kilobase of transcript per million mapped reads (FPKM) values based on read alignments. Gene expression profiles were visualised using boxplots and PCA, while PCC were computed to assess relationships among samples and presented as heatmaps.

To investigate differences in gene expression among samples, differential expression analysis was performed using the R package edgeR (Robinson et al., 2009). Genes with a false discovery rate (FDR) < 0.05 and |Fold Change| > 2 were identified as differentially expressed genes (DEGs). GO annotation was performed for all DEGs, and the number of genes associated with each GO term was quantified. GO terms were classified into the categories Molecular Function, Cellular Component, and Biological Process for visualisation. Enrichment analysis was performed using a hypergeometric test, with terms considered significantly enriched at *p* < 0.05 and enrichment type “over.” The top 20 enriched GO terms, ranked by *p*-value, were presented as bar plots. Similarly, KEGG annotation was performed to assign DEGs to corresponding KEGG pathways and generate functional classification plots. KEGG pathway enrichment was analysed using a hypergeometric test, with pathways considered significantly enriched at *p* < 0.05 and enrichment type “over.” The top 20 enriched pathways, ranked by *p*-value, were visualised as bubble plots. Temporal expression trend analysis of DEGs was performed using the Short Time-series Expression Miner (STEM) algorithm, grouping DEGs into 16 distinct expression profiles (Mu et al., 2024). Hierarchical clustering heatmaps were generated and analysed using OECloud tools (https://cloud.oebiotech.com).

### 2.9 WGCNA

To improve the reliability of the analysis, lowly expressed genes were filtered out using the varFilter function in the R package genefilter. WGCNA was subsequently performed using the R package WGCNA *v*1.71 in R *v*4.2.2. The soft-thresholding power was set to 18, the mergeCutHeight parameter to 0.25, and the minimum module size to 50. Module eigengenes were then calculated, and their correlations with traits were assessed using Pearson’s correlation analysis. Modules were considered significantly associated with traits when the absolute correlation coefficient was ≥ 0.6 and the *p*-value was < 0.05. Modules exhibiting strong trait associations were subjected to KEGG pathway enrichment analysis to infer their potential biological functions. To identify hub genes within these modules, the 50 genes with the highest intra-module connectivity were further analysed to assess their central roles within the coexpression network and their potential regulatory functions.

### 2.10 Multi-omics analysis to elucidate key metabolic pathways

A multi-omics approach was employed to characterise key metabolic pathways, with particular emphasis on the PB and PHST pathways. Correlation analysis between DEGs and DSMs was performed following the method described by Li et al. (2022), using the ggcor R package v0.9.8.1 in R *v*4.2.0. Furthermore, DEG–DSM correlation networks were constructed as described by Bai et al. (2022), utilising the stats package in R *v*3.5.1.

### 2.11 Quantitative real-time PCR analysis for gene expression

To validate the transcriptional expression of genes identified through RNA-seq, 14 core genes were selected for verification (**Supplementary Table S1**) using qRT-PCR. The RNA used for qRT-PCR analysis was extracted from the same samples as those used for RNA sequencing. Total RNA was reverse-transcribed into cDNA with a first-strand cDNA synthesis kit (Vazyme Biotech Co., Ltd., Nanjing, Jiangsu, China) in a 20 μL reaction containing 1 μg of RNA. Primer specificity and PCR amplification products were confirmed by 2% agarose gel electrophoresis. The qRT-PCR programme comprised an initial denaturation at 95°C for 30 s, followed by 40 cycles of 95°C for 5 s and 60°C for 30 s for annealing and extension (Tianlong Technology Co., Ltd., Xi’an, China). Gene expression levels were normalised to the reference gene 60S Ribosomal Protein L8 (*SsRPL8*). All assays included three biological replicates, each with three technical replicates. Relative expression levels were calculated using the 2^−ΔΔCt^ method (Livak and Schmittgen, 2000).

RNA-seq data and qRT-PCR data for each gene were used to calculate the normalized log_2_ fold change, and Ss4 data were used as a reference. Then, linear fitting of the RNA-seq data and qRT-PCR data was performed, and the correlation index (R^2^) was calculated.

## 3 Results

### 3.1 Seasonal characteristics of cambial activity in *S. superba*

*S. superba* is an evergreen broad-leaved tree species whose vascular cambium exhibits distinct, stage-specific activity patterns across different seasons (**Figure 1**). Based on previous studies on the seasonal dynamics of cambial activity in woody plants (Guo et al., 2022; Shi et al., 2021; Xi and Zhao, 2012; Zeng et al., 2025; Zhong et al., 2011), cambial activity in *S. superba* was classified into four stages: onset of spring cambial activity (April), cambial active period (June and August), decline in cambial activity (October), and onset of cambial dormancy (December). Cambial samples collected at these representative time points capture the dynamic transition of cambial activity from an active to a dormant state. In spring, the cambium gradually resumes activity and enters the initiation phase of cell division and growth. During summer, the cambium remains highly active, representing the most vigorous growth period of the year. As autumn progresses, cambial activity gradually declines, whereas in winter, cambial activity is markedly reduced, entering a relatively quiescent state. Collectively, these seasonal differences in cambial activity provide a clear biological framework for investigating internal metabolic changes associated with different cambial activity stages.

### 3.2 Untargeted metabolomics analysis were conducted to characterise metabolic changes

To investigate internal alterations across different seasonal cambial activity stages, metabolomic analyses were initially conducted. To evaluate the stability and reproducibility of MS measurements, total ion chromatograms (TICs) from multiple QC samples were overlaid. The TICs exhibited a high degree of concordance in both retention time and peak intensity (**Supplementary Figures S1A,B**), indicating consistent MS signals across runs and thereby confirming the reliability of subsequent metabolomic analyses. This reproducibility was further validated by PCCs, with values among QC samples ≥ 0.9998 (**Supplementary Figures S1C,D**). Furthermore, coefficient of variation (CV) analysis revealed that over 85% of metabolites in the QC samples displayed CVs < 0.3 (**Supplementary Figures S1E,F**), demonstrating the robustness and consistency of the dataset. PCA was performed on all samples, including QC samples, to assess variation in metabolite profiles across treatments and within groups. The analysis revealed clear separation between treatment groups, indicating significant differences in metabolic composition, while samples within each group clustered closely, reflecting high intra-group reproducibility (**Supplementary Figure S2A**). The first five principal components collectively explained over 75% of the total variance (**Supplementary Figure S2B**), confirming that the PCA model reliably represented the underlying structure of metabolic differences among the samples.

A total of 3,024 metabolites were identified, including 2,041 in positive ion mode and 983 in negative ion mode (**Supplementary Table S2**). These metabolites were predominantly classified into amino acids and derivatives (637), organic acids (432), benzene and substituted derivatives (386), alcohols and amines (128), flavonoids (127), alkaloids (124), phenolic acids (117), terpenoids (105), and heterocyclic compounds (103) (**Supplementary Figure S3**). PCA was performed on all grouped samples to assess inter-group variation and intra-group consistency. The results revealed clear separation among treatment groups in the principal component space, indicating pronounced differences in metabolic composition, whereas samples within each group clustered tightly, demonstrating high experimental reproducibility (**Supplementary Figure S4**). Building on these observations, supervised modelling analyses were conducted using PLS-DA and OPLS-DA, which revealed distinct separation between treatment groups in the model space (**Supplementary Figures S5, and S6**). With the exception of the Ss12_*vs*_Ss6 comparison (Q² = 0.351), Q² values for all other comparisons ranged from 0.528 (Ss6_*vs*_Ss4) to 0.952 (Ss12_*vs*_Ss8) (**Supplementary Figure S7**), demonstrating strong predictive performance of the models and further confirming significant differences in metabolite composition across treatment conditions.

**Figure 2:**
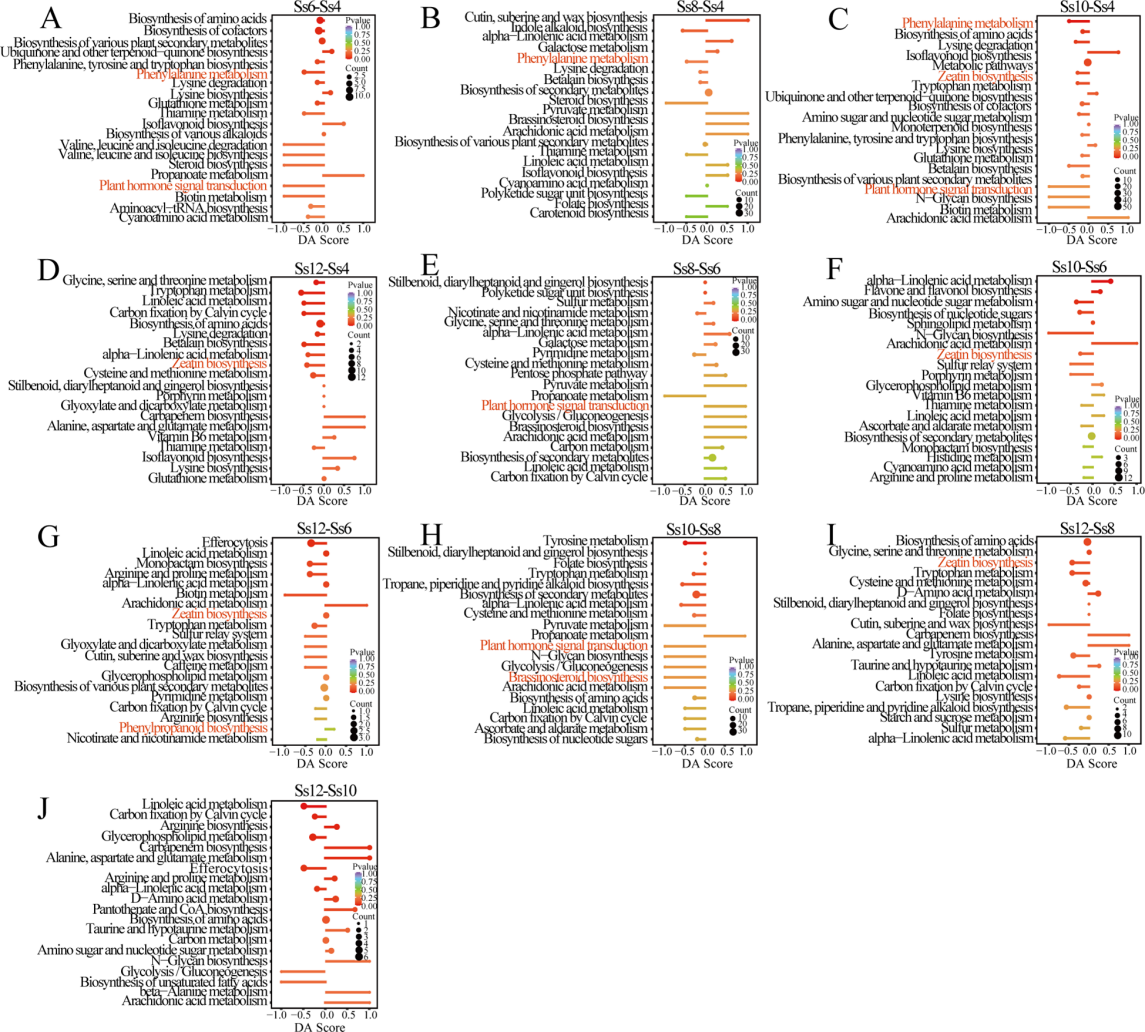
Differential abundance (DA) scores of differentially synthesised metabolites (DSMs). **(A)** Ss6_*vs*_Ss4; **(B)** Ss8_*vs*_Ss4; **(C)** Ss10_*vs*_Ss4; **(D)** Ss12_*vs*_Ss4; **(E)** Ss8_*vs*_Ss6; **(F)** Ss10_*vs*_Ss6; **(G)** Ss12_*vs*_Ss6; **(H)** Ss10_*vs*_Ss8; **(I)** Ss12_*vs*_Ss8; and **(J)** Ss12_*vs*_Ss10. The *y*-axis represents the names of the differential pathways, and the *x*-axis represents the DA score, which reflects the overall changes of all metabolites within a given pathway. A DA score of “1” indicates that all identified metabolites in the pathway are upregulated, whereas a DA score of “–1” indicates that all identified metabolites are downregulated. The length of each line segment represents the absolute value of the DA score. The size of the circle at the end of each line indicates the number of differential metabolites in that pathway. Circles positioned to the left of the central axis with longer line segments indicate a stronger tendency towards downregulation, whereas circles positioned to the right with longer line segments indicate a stronger tendency towards upregulation. Circle size is proportional to the number of metabolites. The colour of the line segments and circles reflects the *p*-value, with red indicating smaller *p*-values and purple indicating larger *p*-values.

Among the ten comparison groups, Ss12_*vs*_Ss8 exhibited the highest number of DSMs (1,156), followed by Ss12_*vs*_Ss4 (1,072), Ss8_*vs*_Ss4 (1,021), and Ss10_*vs*_Ss8 (1,004), whereas Ss10_*vs*_Ss6 showed the fewest, with only 354 DSMs (**Supplementary Figure S8**). These results indicate pronounced differences in metabolic pathway enrichment across all stages, primarily involving amino acid metabolism, fatty acid metabolism, phenylalanine metabolism, PB, plant hormone biosynthesis and signalling (including zeatin and brassinosteroid pathways), carbohydrate metabolism, and secondary metabolite biosynthesis (**Figure 2**).

**Figure 3:**
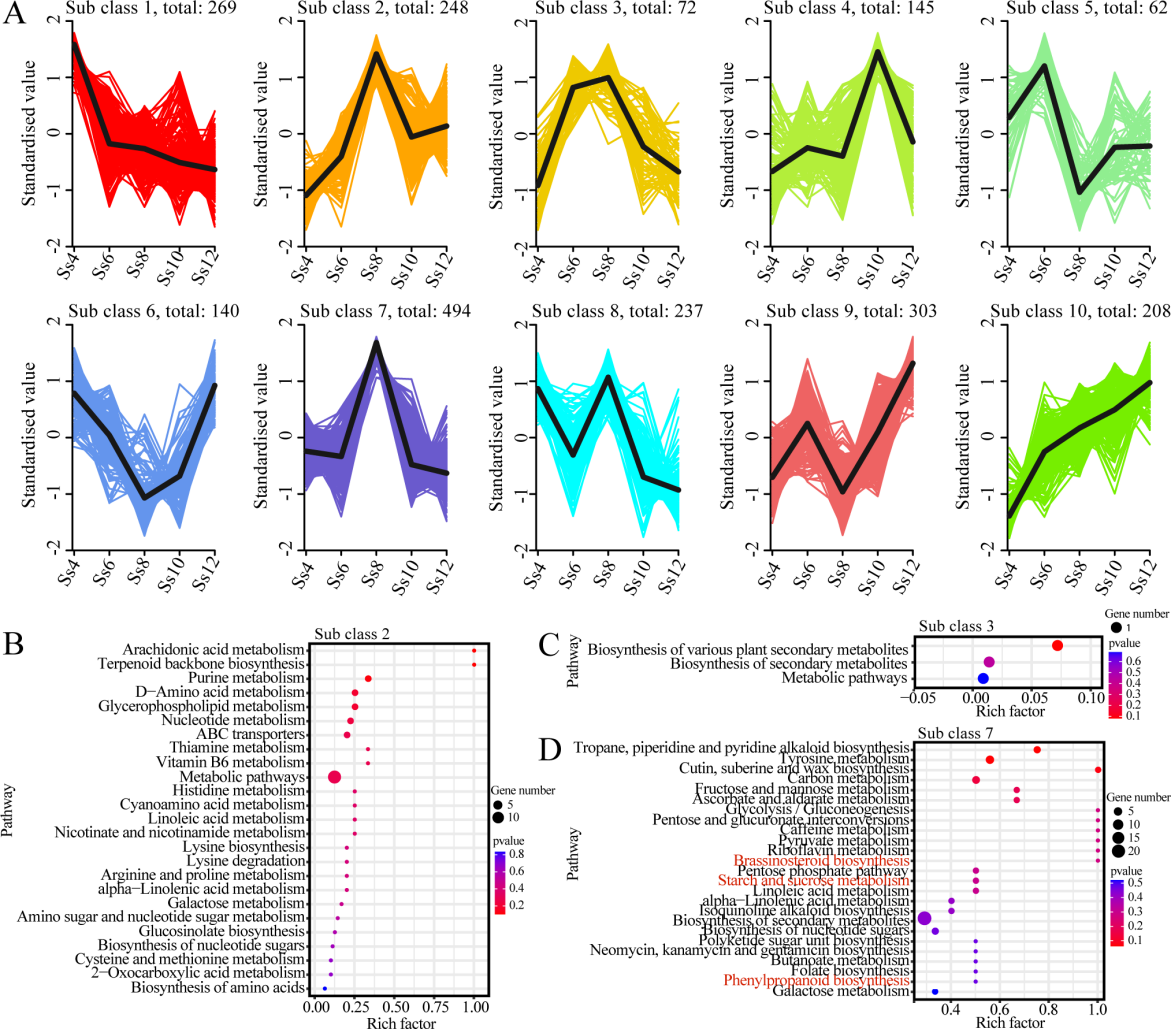
K-means analysis of DSMs. **(A)** K-means clustering of DSMs. The *x*-axis represents sample groups, and the *y*-axis shows the normalised relative abundance of metabolites. Subclass indicates the cluster number of metabolites sharing a similar expression pattern, and total denotes the number of metabolites in that cluster. KEGG enrichment analysis of DSMs is shown for subclass 2 **(B)**, subclass 3 **(C)**, and subclass 7 **(D)**.

To investigate metabolite abundance patterns across all stages, K-Means clustering was performed on 2,178 DSMs identified across all comparison groups, yielding ten distinct subclasses (**Figure 3A**). Each subclass represents a unique dynamic pattern of metabolite variation across the seasonal cambial activity stages. Notably, DSMs in Subclasses 2, 3, and 7 were significantly upregulated at the Ss6 or Ss8 stages, followed by a gradual decline, exhibiting clear stage-specific enrichment patterns (**Figure 3A**). To further elucidate the potential functions of metabolites with distinct expression profiles, KEGG pathway enrichment analyses were performed for these three representative subclasses. DSMs in Subclass 2 were predominantly enriched in arachidonic acid metabolism, terpenoid and flavonoid biosynthesis, lipid metabolism, vitamin metabolism, and various amino acid metabolic pathways (**Figure 3B**). Subclass 3 DSMs were mainly associated with plant secondary metabolite biosynthesis pathways (**Figure 3C**). In Subclass 7, DSMs were enriched in alkaloid biosynthesis, carbohydrate metabolism (including starch and sucrose metabolism, fructose and mannose metabolism, glycolysis/gluconeogenesis, pentose and glucuronate interconversions, and pentose phosphate pathways), phenylpropanoid and lignin biosynthesis, and brassinosteroid biosynthesis (**Figure 3D**). These findings indicate that the cambium of *S. superba* dynamically accumulates metabolites with stage-specific functions.

### 3.3 Characterisation of small-molecule metabolites related to lignin biosynthesis

The MRM metabolite analysis conducted in dual-ion mode revealed dense signal peaks with clear and symmetrical shapes (**Supplementary Figure S9**), indicating that the MS system exhibited high separation efficiency and detection sensitivity, thereby ensuring stable and reliable data quality. To further assess measurement stability, TICs from different QC samples were overlaid, revealing a high degree of consistency in both retention time and signal intensity across the QC curves (**Supplementary Figure S10**). These results demonstrate that the MS provided excellent reproducibility and signal stability when analysing the same sample at different time points, thereby supporting the reliability of subsequent quantitative metabolite analyses.

**Figure 4:**
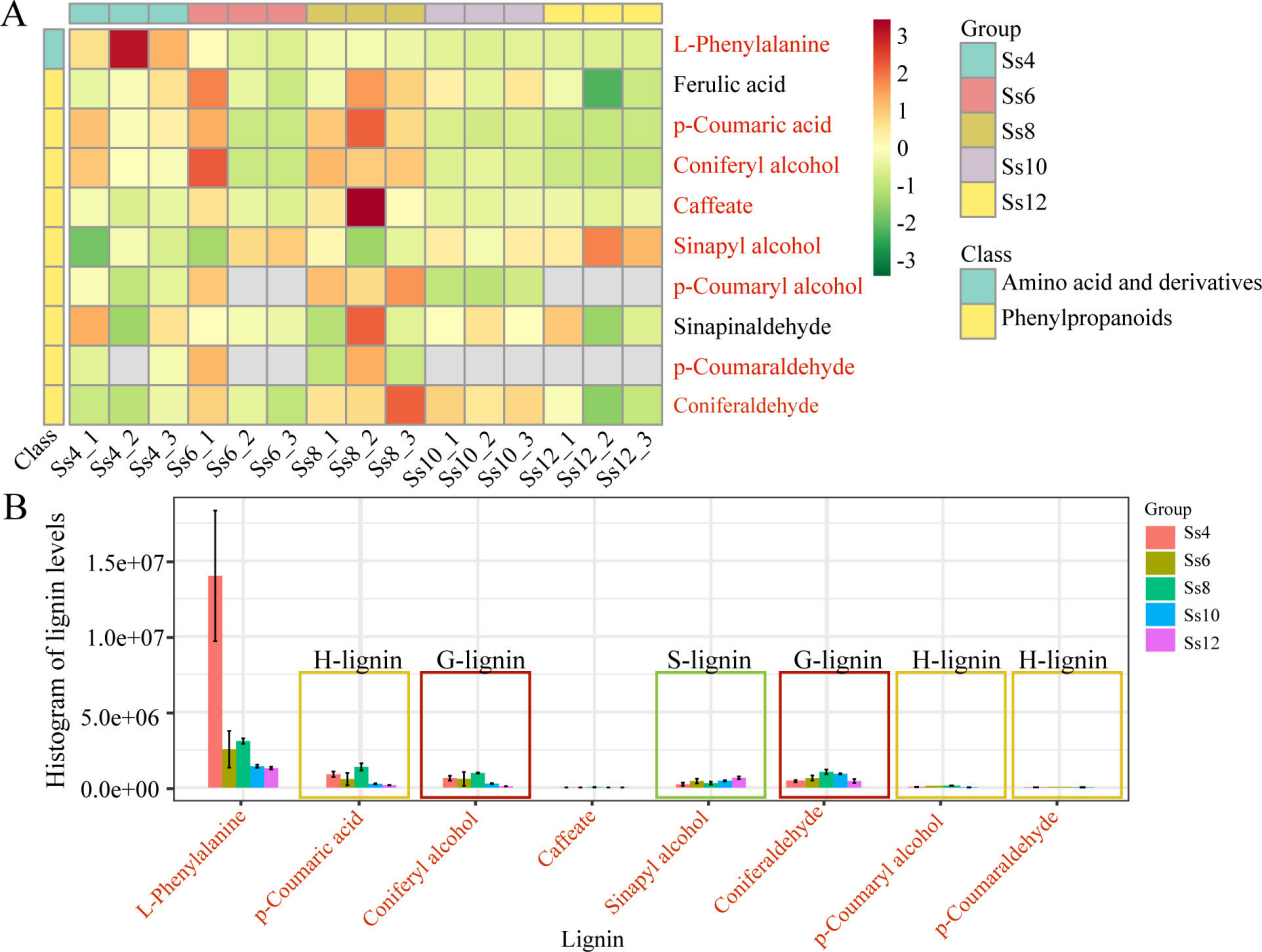
Analysis of metabolites in the lignin biosynthesis pathway. **(A)** Cluster analysis of metabolites detected in the lignin pathway, with DSMs highlighted in red. **(B)** Histogram showing the levels of lignin-related DSMs.

A total of 10 lignin-related metabolites were identified in the analysis of lignin biosynthesis-related small molecules (**Supplementary Table S3**), with 9 detected in negative ion mode and 1 in positive ion mode (**Supplementary Table S3**). Among these metabolites, 1 was classified as an amino acid and its derivatives, while the remaining 9 were phenylpropanoids (**Supplementary Table S3**). Further analysis revealed 8 DSMs associated with the lignin biosynthesis pathway (**Figure 4A**). The highest number of DSMs (6) was observed in the Ss12_*vs*_Ss8 comparison group, followed by Ss10_*vs*_Ss4 and Ss10_*vs*_Ss8, whereas only 2 were detected in Ss8_*vs*_Ss6, Ss12_*vs*_Ss6, and Ss12_*vs*_Ss10 (**Supplementary Table S4**). Among these metabolites, sinapyl alcohol, an intermediate of S-lignin, displayed an upregulated trend, while L-phenylalanine was downregulated. The remaining metabolites, including intermediates of G/H-lignin, exhibited a dynamic pattern characterised by an initial increase followed by a decline, with peak accumulation at the Ss8 stage (**Figures 4A, B**). These metabolites exhibited distinct stage-specific dynamics across the seasonal cambial activity stages, revealing the temporal regulation of metabolic flux in lignin biosynthesis.

### 3.4 RNA-seq-based transcriptomic analysis revealed gene expression dynamics in the vascular cambium

To explore gene expression across the seasonal cambial activity stages in *S. superba*, RNA-seq was performed on samples from five stages. A total of 666.956 million raw reads were generated from 15 samples, yielding 666.776 million clean reads after filtering (**Table 1**). The proportion of Q30 bases ranged from 96.68% to 97.15%, and GC content varied between 44.06% and 45.36% (**Table 1**), indicating high-quality sequencing data. *De novo* assembly produced 118,426 unigenes with a total length of 136,739,149 bp, an average length of 1,154.64 bp, and an N50 of 1,616 bp (**Supplementary Tables S5, S6**). Mapping rates to the reference genome ranged from 86.61% to 89.36% (**Supplementary Table S7**), confirming the reliability of the assembly. Functional annotation of 18,750 unigenes (15.83%) using the NR protein database identified kiwifruit (*Actinidia chinensis* var. *Chinensis*, 52.08%) as the closest match, followed by grapes (*Vitis vinifera*, 5.99%) and cork oak (*Quercus suber*, 2.69%) (**Supplementary Figure S11**). GO annotation classified 9,104 unigenes (7.69%) into three categories: cellular component, molecular function, and biological process, with the most abundant terms being cellular process, metabolic process, and single-organism process (**Supplementary Figure S12A**). KEGG annotation assigned 8,505 unigenes (7.18%) to pathways related to metabolism, genetic information processing, environmental information processing, and cellular processes (**Supplementary Figure S12B**), with metabolism containing the largest number of genes. Furthermore, 425 TFs representing 47 families were identified, of which *SsERF* (ETH response factor, 36 members) was the most abundant, followed by *SsbHLH* (basic Helix-Loop-Helix, 31), *SsC2H2* (C2H2-type zinc finger, 29), *SsMYB* (28), *SsGRAS* (gibberellin-insensitive (GAI), repressors of ga1-3 (RGA) and scarecrow (SCR)) (24), and *MYB-relate*d (23) (**Supplementary Tables S8, S9**).

**Table 1:** Statistics of clean data.

Sample	Raw reads	Raw bases	Clean reads	Clean bases	Clean ratio (%)	Q20 (%)	Q30 (%)	GC (%)
Ss4_1	42,194,700	6,329,205,000	42,182,732	6,291,933,046	99.97	99.02	96.78	45.36
Ss4_2	44,703,936	6,705,590,400	44,691,400	6,673,513,727	99.97	99.12	97.15	45.12
Ss4_3	39,464,306	5,919,645,900	39,453,534	5,890,511,400	99.97	99.10	97.10	45.22
Ss6_1	46,867,650	7,030,147,500	46,855,384	7,000,778,301	99.97	99.00	96.77	45.14
Ss6_2	41,260,072	6,189,010,800	41,249,216	6,158,440,121	99.97	99.09	97.04	45.07
Ss6_3	41,874,962	6,281,244,300	41,863,614	6,255,154,936	99.97	99.00	96.77	45.10
Ss8_1	40,874,836	6,131,225,400	40,863,414	6,098236,836	99.97	98.99	96.74	44.55
Ss8_2	45,284,210	6,792,631,500	45,271,068	6,762,437,919	99.97	99.06	96.99	44.79
Ss8_3	63,927,494	9,589,124,100	63,909,484	9,552,043,424	99.97	99.03	96.88	44.80
Ss10_1	41,347,392	6,202,108,800	41,336,450	6,174,809,150	99.97	98.97	96.68	44.26
Ss10_2	41,179,606	6,176,940,900	41,168,832	6,154,830,293	99.97	99.11	97.07	44.17
Ss10_3	42,514,116	6,377,117,400	42,502,622	6,347,392,275	99.97	99.08	97.00	44.22
Ss12_1	39,514,814	5,927,222,100	39,503,910	5,895,005,676	99.97	99.10	97.09	44.21
Ss12_2	42,667,516	6,400,127,400	42,655,404	6,367,493,826	99.97	98.99	96.76	44.06
Ss12_3	53,283,226	7,992,483,900	53,268,906	7,949,638,811	99.97	99.05	96.96	44.22

The median gene expression levels across all samples were comparable, with similar interquartile ranges (IQRs), indicating consistent sequencing depth and expression distribution (**Supplementary Figure S13A**). The peaks of the expression distributions were also broadly similar across samples, with overall curve heights and positions closely aligned, further suggesting uniform sequencing depth and gene expression (**Supplementary Figure S13B**). Pearson correlation analysis revealed strong correlations between samples, with R² values ≥ 0.972 (**Supplementary Figure S13C**). Samples within the same group clustered tightly, whereas clear separation was observed between groups (**Supplementary Figure S13D**), confirming the appropriateness of sample selection and high biological reproducibility.

**Figure 5:**
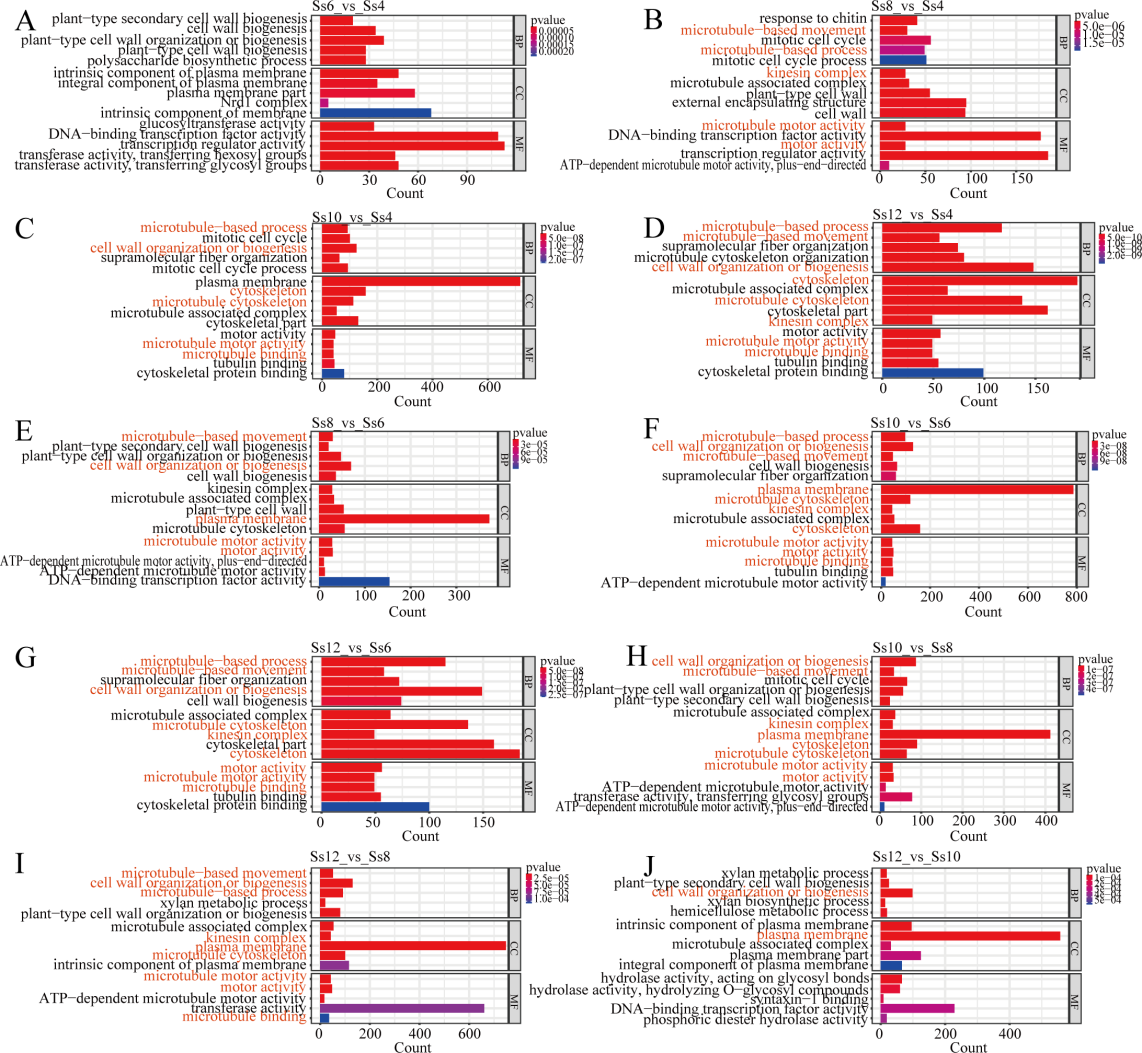
GO enrichment analysis of differentially expressed genes (DEGs). **(A)** Ss6_*vs*_Ss4; **(B)** Ss8_*vs*_Ss4; **(C)** Ss10_*vs*_Ss4; **(D)** Ss12_*vs*_Ss4; **(E)** Ss8_*vs*_Ss6; **(F)** Ss10_*vs*_Ss6; **(G)** Ss12_*vs*_Ss6; **(H)** Ss10_*vs*_Ss8; **(I)** Ss12_*vs*_Ss8; and **(J)** Ss12_*vs*_Ss10.

**Figure 6:**
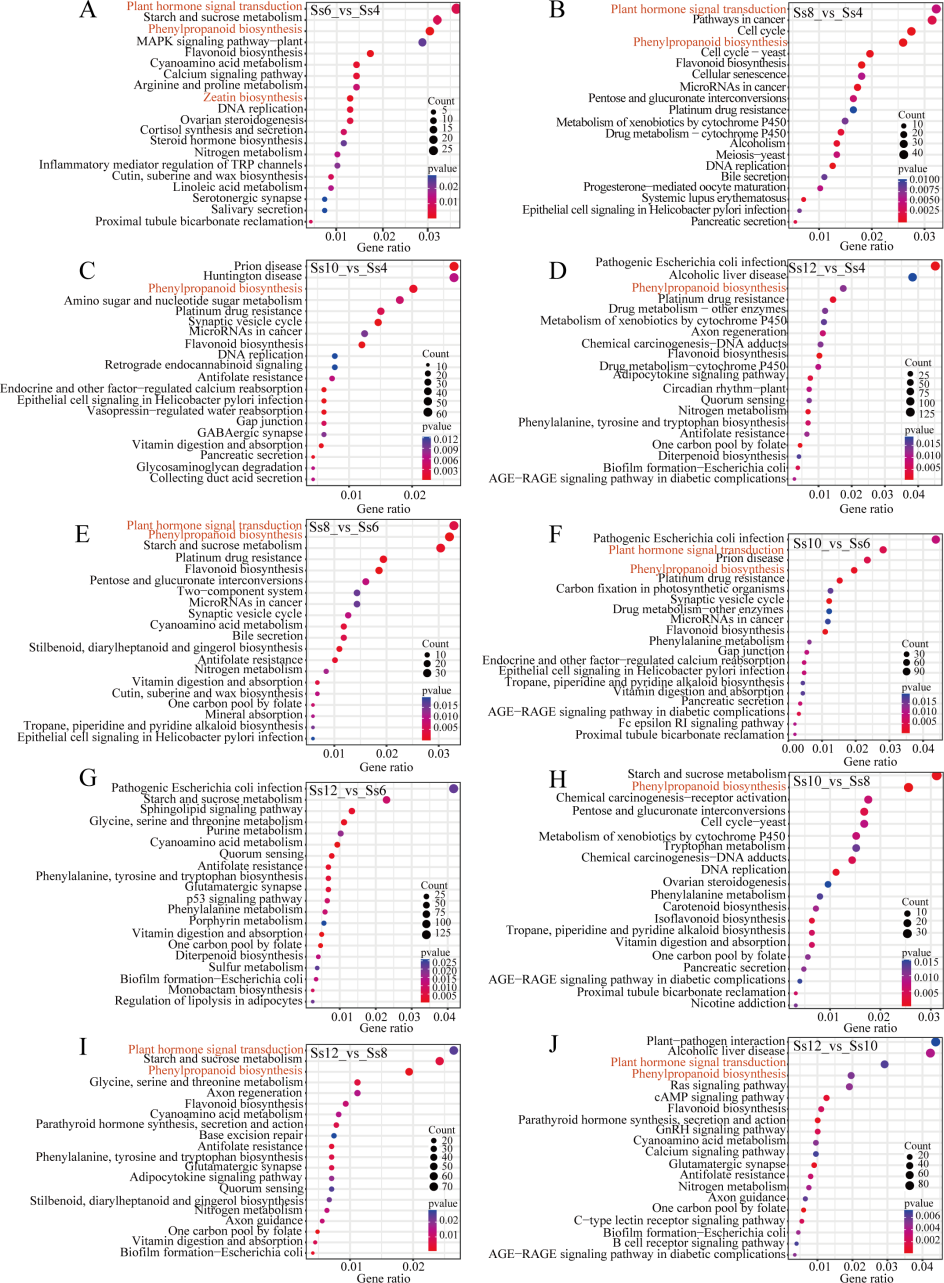
KEGG enrichment analysis of DEGs. **(A)** Ss6_*vs*_Ss4; **(B)** Ss8_*vs*_Ss4; **(C)** Ss10_*vs*_Ss4; **(D)** Ss12_*vs*_Ss4; **(E)** Ss8_*vs*_Ss6; **(F)** Ss10_*vs*_Ss6; **(G)** Ss12_*vs*_Ss6; **(H)** Ss10_*vs*_Ss8; **(I)** Ss12_*vs*_Ss8; and **(J)** Ss12_*vs*_Ss10.

A total of 19,610 DEGs were identified across the 10 comparison groups, showing significant expression differences between at least two libraries (**Supplementary Tables S10, S11**). The highest number of DEGs was observed in the Ss12_*vs*_Ss6 comparison (13,813 DEGs), followed by Ss12_*vs*_Ss4 (12,522) and Ss12_*vs*_Ss8 (12,196), while Ss6_*vs*_Ss4 yielded the fewest (2,738) (**Supplementary Table S11**). GO enrichment analysis revealed that DEGs from several comparison groups were significantly enriched in terms associated with microtubule dynamics and cytoskeletal remodelling, including “microtubule motor activity” (8), “motor activity” (6), “microtubule binding” (5), “cell wall organization or biogenesis” (8), “microtubule-based movement” (7), “microtubule-based process” (6), “kinesin complex” (6), “microtubule cytoskeleton” (6), “cytoskeleton” (5), and “plasma membrane” (5) (**Figure 5**). KEGG enrichment analysis further revealed that DEGs in Ss6_*vs*_Ss4, Ss8_*vs*_Ss4, Ss8_*vs*_Ss6, Ss10_*vs*_Ss6, Ss12_*vs*_Ss8, and Ss12_*vs*_Ss10 were significantly enriched in pathways related to PHST and PB (**Figures 6A, B, E, F, I, J**). Similarly, the PB pathway was significantly enriched in Ss10_*vs*_Ss4, Ss12_*vs*_Ss4, and Ss10_*vs*_Ss8 (**Figures 6C, D, H**). In addition, 239 differentially expressed TFs were identified across the 10 comparison groups, predominantly from families such as *SsERF* (23 genes), *SsbHLH* (18), *SsC2H2* (16), *SsMYB* (15), *SsB3* (12), *SsbZIP* (basic leucine zipper, 12), *SsGRAS* (12), *SsMYB-related* (9), *SsWRKY* (9), *SsC3H* (C3H-type zinc finger, 8), *SsNA*C (8), *SsNin-like* (NODULE INCEPTION-like, 8), *SsDof* (DNA binding with one finger, 7), and *SsFAR1* (far-red impaired response 1, 7) (**Supplementary Table S12**). HCA of these DEGs revealed stage-specific transcriptomic profiles across seasonal cambial activity of *S. superba* (**Supplementary Figure S14**), providing an essential foundation for further temporal progression analysis.

**Figure 7:**
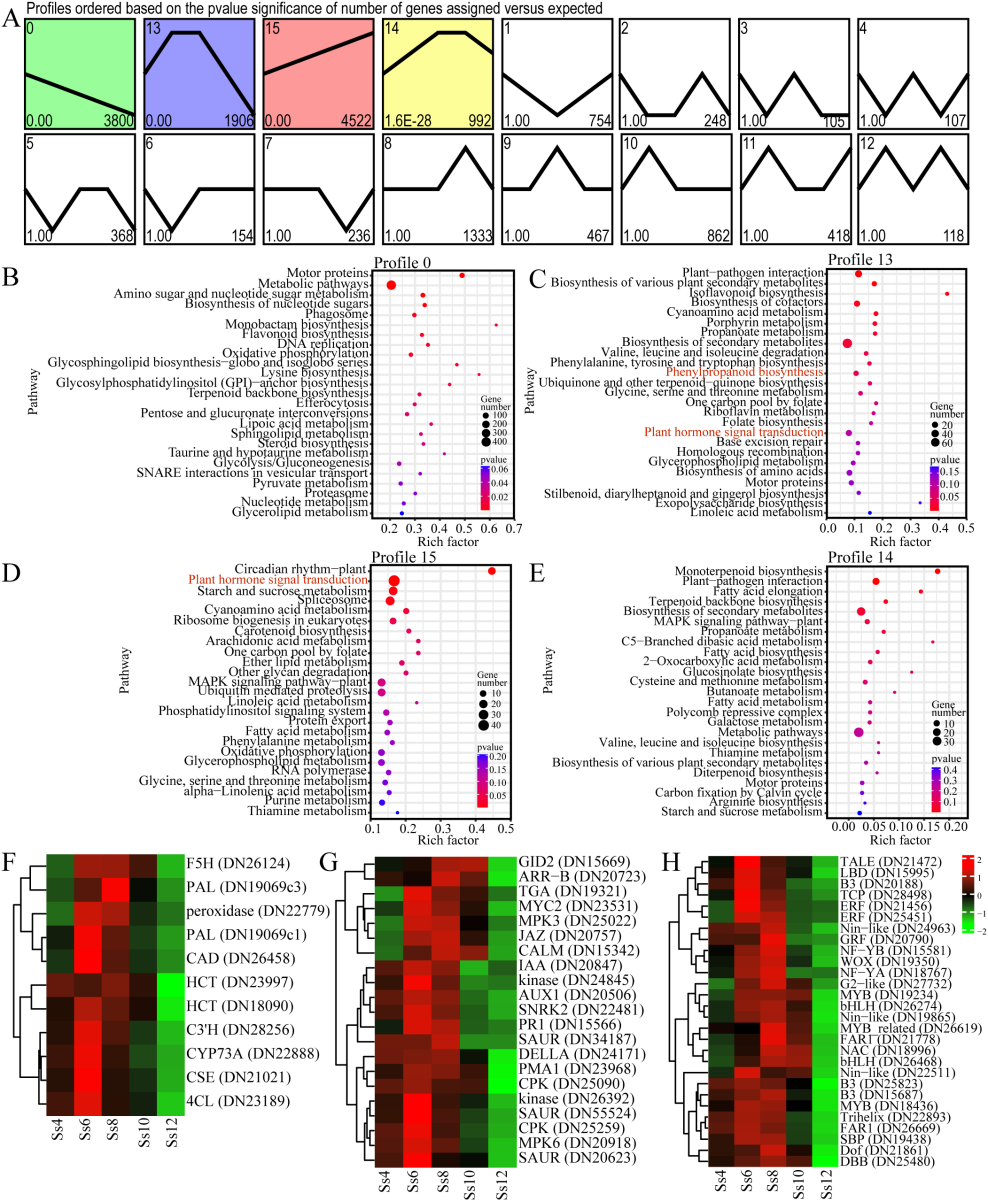
Trend analysis of DEGs. **(A)** Overall trend analysis, with profiles ordered based on the significance of the number of genes assigned versus the expected number (*p*-value). KEGG enrichment analysis is shown for Profile 0 **(B)**, Profile 13 **(C)**, Profile 15 **(D)**, and Profile 14 **(E)**. **(F)** Phenylpropanoid biosynthesis (PB)-related DEGs in Profile 13. **(G)** Plant hormone signal transduction (PHST)-related DEGs in Profile 13. **(H)** Differentially expressed transcription factors in Profile 13.

To identify representative gene clusters with distinct expression patterns across seasonal cambial activity of *S. superba*, trend analysis was performed on the DEGs. A total of 16,390 DEGs were assigned to 16 expression profiles, with four main expression trends (*p* ≤ 0.05) broadly grouped into three categories: continuous upregulation (profile 15), continuous downregulation (profile 0), and initial upregulation followed by downregulation (profiles 0 and 14) (**Figure 7A**). KEGG enrichment analysis revealed that genes in profile 13 were significantly enriched in PB and PHST pathways (**Figures 7B−E**). Specifically, 11 PB-related genes were identified, including *Ss4CL*, *SsF5H* (ferulate 5-hydroxylase), *SsCAD* (cinnamyl alcohol dehydrogenase), *SsPAL* (phenylalanine ammonia-lyase), *SsHCT* (hydroxycinnamoyl-CoA shikimate), *SsCYP73A* (cinnamate 4-hydroxylase), *SsC3’H* (quinate 3′-hydroxylase), *peroxidase*, and *SsCSE* (caffeoyl shikimate esterase). In addition, 21 PHST-associated genes encompassed multiple signalling pathways: AUX (*SsAUX1*, *SsIAA*, *2 Sskinase*, *SsMPK3* (mitogen-activated protein kinase 3), and 3 *SsSAURs* (small AUX up RNA)), phytosulfokine (PSK) (*SsCALM* (calmodulin), 2 *SsCPKs* (calcium-dependent protein kinases), and *SsPMA1* (plasma membrane H^+^-ATPase 1)), GA (*SsDELLA* and *SsGID2* (GA-insensitive dwarf 2)), jasmonic acid (JA; *SsJAZ* (jasmonate ZIM-domain protein) and *SsMYC2*), salicylic acid (SA; *SsPR1* (pathogenesis-related protein 1) and *SsTGA)*, abscisic acid (ABA; *SsSNRK2* (SNF1-related protein kinase 2)), CTK (*SsARR-B* (type-B *Arabidopsis* response regulator)), and ETH (*SsMPK6*) (**Figures 7F, G**). These genes exhibited an expression pattern characterised by initial upregulation followed by downregulation, peaking in June or August (**Figures 7F, G**). Furthermore, 28 differentially expressed TFs within this profile, including *SsMYB* (3), *SsNin-like* (3), *SsB3* (3), *SsbHLH* (2), *SsERF* (2), and *SsFAR1* (2), exhibited similar temporal dynamics, also peaking in June or August (**Figure 7H**). These dynamically expressed structural genes and TFs likely act synergistically to coregulate stage-specific cambial activity.

### 3.5 WGCNA identified core candidate genes potentially regulating vascular cambium activity and differentiation

**Figure 8:**
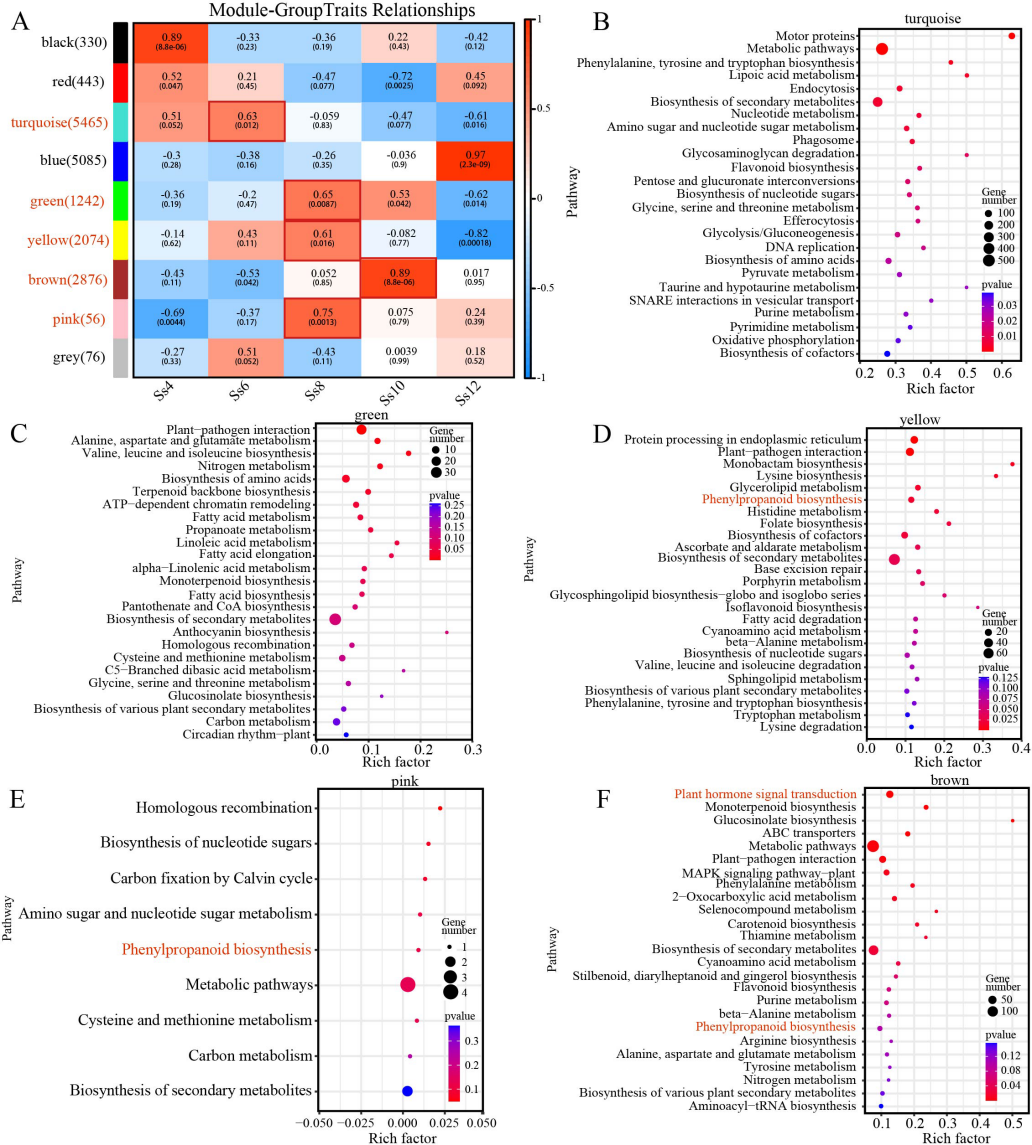
WGCNA identified core candidate genes potentially regulating vascular cambium activity and differentiation. **(A)** Heatmap of trait-associated modules. KEGG enrichment analysis of genes in the Turquoise **(B)**, Green **(C)**, Yellow **(D)**, Pink **(E)**, and Brown **(F)** modules.

**Figure 9:**
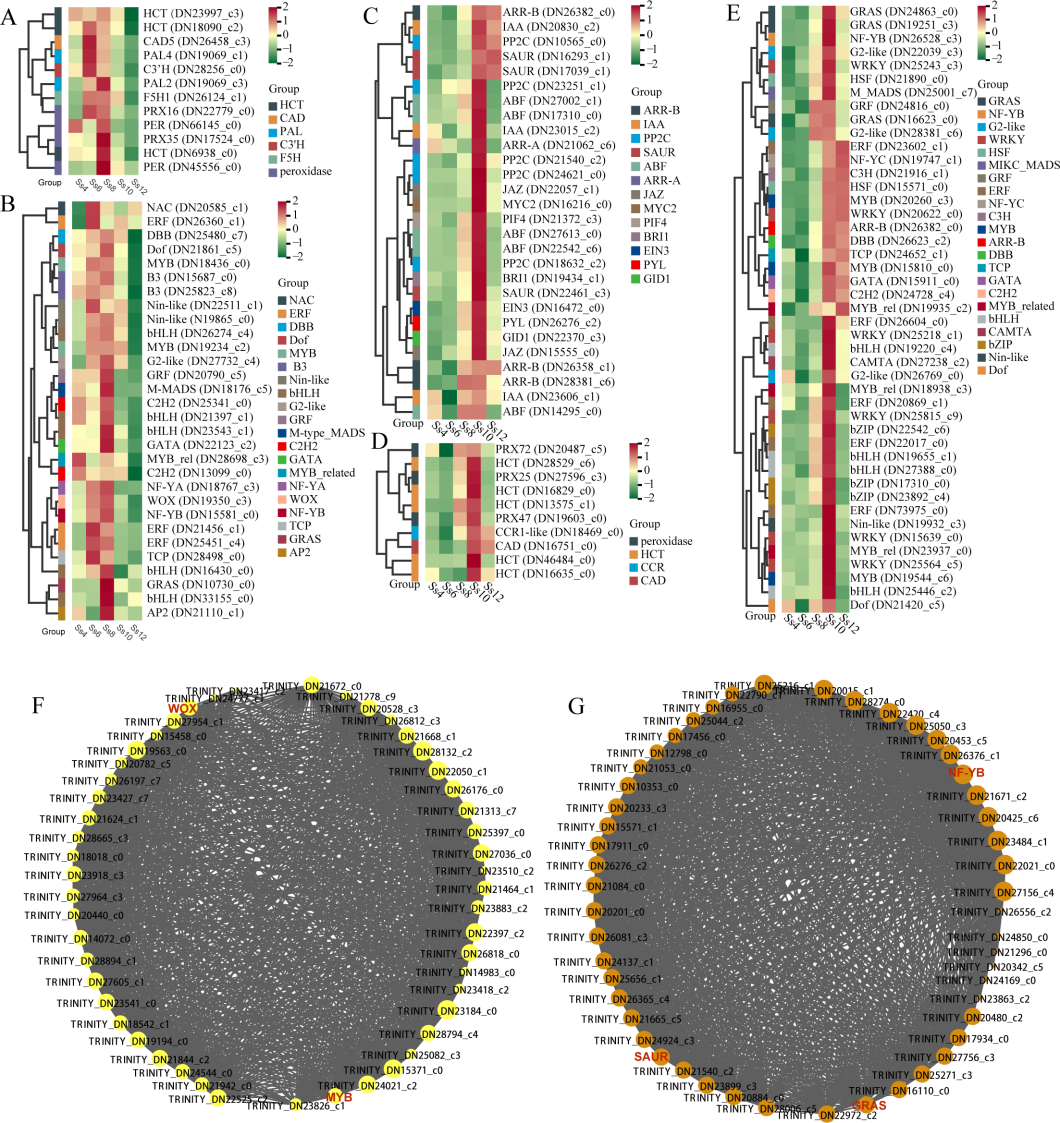
Top 50 hub gene analysis. Hierarchical clustering heatmaps of PB-related genes **(A)** and transcription factors (TFs) **(B)** in the yellow module; PHST-related genes **(C)**, PB-related genes **(D)**, and TFs **(E)** in the brown module. **(F)** Network of the top 50 genes in the yellow module; **(G)** network of the top 50 genes in the brown module. In the network diagrams, edges represent the strength of interactions between genes, with genes connected to more nodes occupying more central positions in the network.

To further identify potential regulatory modules and core genes in the cambium of *S. superba*, a WGCNA was performed based on the DEGs. In total, 17,647 genes were classified into 9 coexpression modules, with the Grey module comprising genes not assigned to any other module (**Figure 8A**; **Supplementary Table S13**). Correlation analysis revealed that the Turquoise module was significantly positively associated with the Ss6 stage, the Pink, Yellow, and Green modules with the Ss8 stage, and the Brown module with the Ss10 stage (**Figure 8A**). KEGG pathway enrichment analysis of DEGs within these stage-associated modules indicated that the Pink and Yellow modules were enriched in the PB pathway, whereas the Brown module was enriched in both PHST and PB pathways (**Figures 8B−F**). Within the yellow module, 12 PB-related genes were identified, including 4 *peroxidases*, 3 *SsHCT*s, 2 *SsPAL*s, 1 *SsCAD*, 1 *SsC3’H*, and 1 *SsF5H* (**Figure 9A**), along with 30 TFs, such as 5 *SsbHLH*s, 3 *SsERF*s, 2 *SsMYB*s, 2 *SsC2H2*s, and 2 *SsB3*s (**Figure 9B**). These genes exhibited a dynamic expression pattern, characterised by an initial upregulation followed by a decline, peaking in either June or August (**Figures 9A, B**). In the pink module, only a single PB-related gene, *SsCAD*, was identified (**Figure 8E**). By contrast, the Brown module contained a larger set of genes: 28 PHST-related genes, including 5 *SsABF*s (ABA-responsive element binding factors), 5 *SsPP2C*s (protein phosphatase 2C), 3 *SsSAUR*s, 3 *SsIAA*s, and 3 *SsARR-B*s (**Figure 9C**); 10 PB-related genes, including 5 *SsHCT*s, 3 *peroxidases*, *SsCAD*, and *SsCCR* (cinnamoyl-CoA reductase) (**Figure 9D**); and 45 TFs, such as 6 *SsWRKY*s, 6 *SsERF*s, 4 *SsbHLH*s, 3 *SsbZIP*s, 3 *SsG2-like*, 3 *SsGRAS*s, and 3 *SsMYB*s (**Figure 9E**). Genes in this module generally exhibited a gradual decline in expression, peaking primarily in October (**Figures 9C−E**). Further functional annotation of the top 50 genes ranked by connectivity within each coexpression module identified several key TFs and signalling genes potentially involved in the regulation of cambial activity in *S. superba*. In the yellow module, PB-related core TFs, such as *SsMYB* and *SsWOX*, were identified (**Figure 9F**). In the brown module, PB- and PHST-related core TFs, including *SsGRAS*, *SsSAUR*, and *SsNF-YB* (nuclear factor Y, subunit B), were found (**Figure 9G**). These results provide valuable insights into the transcriptional regulation mechanisms driving cambial activity, specifically highlighting several regulatory genes and TFs associated with PB and PHST.

### 3.6 Integrated transcriptomic and metabolomic analyses identified key regulatory nodes in the PB pathways

**Figure 10:**
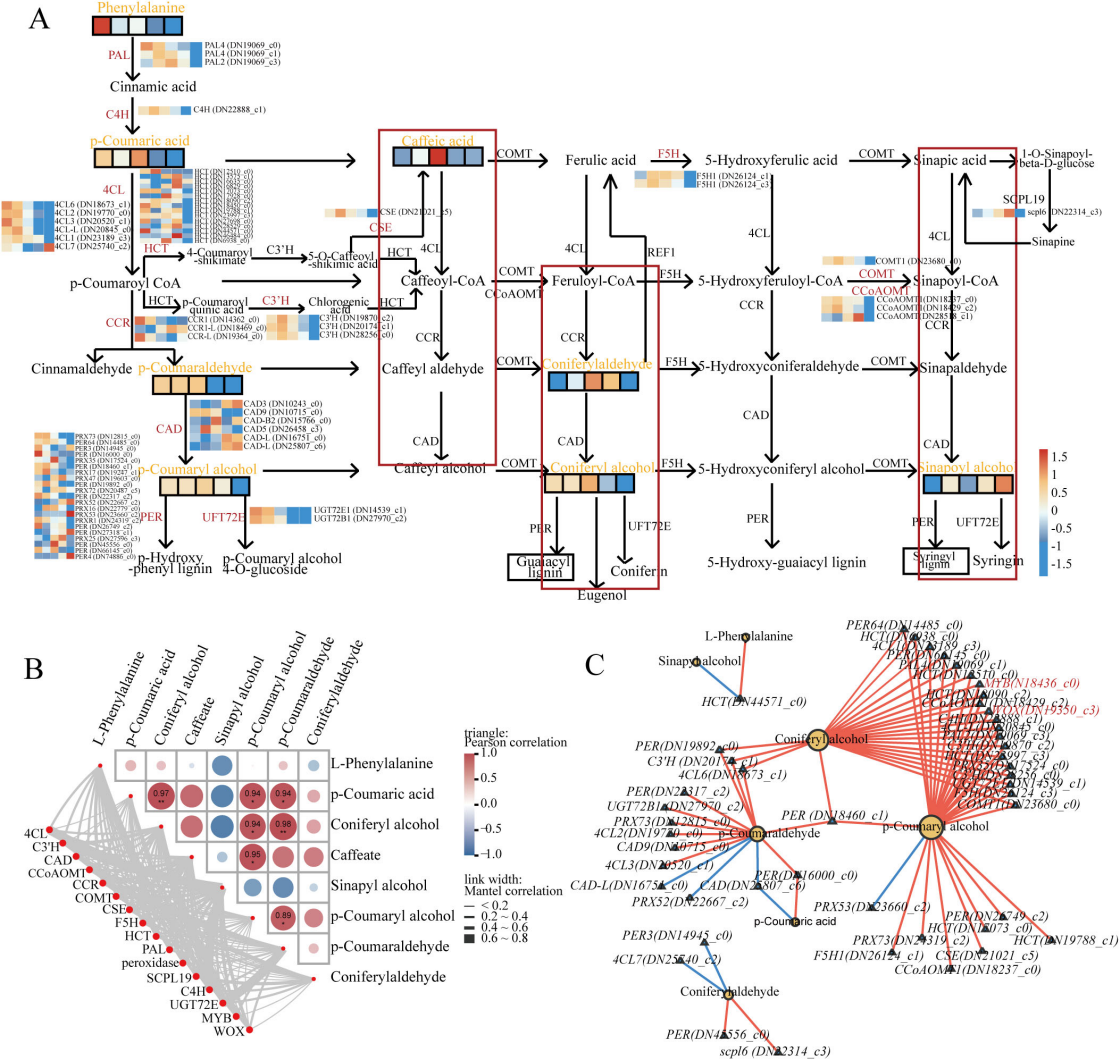
Analysis of DEGs and DSMs in the PB pathway. **(A)** DEGs and DSMs mapped onto the PB pathway. **(B)** Correlation analysis between DSMs and DEGs. Lines in the lower-left indicate Mantel test results, with line thickness representing the overall correlation coefficient. The triangular heatmap displays correlations among metabolites, with cell colours reflecting correlation strength (red: positive; blue: negative). **(C)** Correlation network of DEGs and DSMs. Node size represents connectivity (i.e., the number of significantly correlated metabolites), and edge colour denotes the type of correlation (red: positive; blue: negative).

In the PB pathway, 68 DEGs were identified, including 14 structural genes (**Figure 10A**; **Supplementary Table S14**). These genes exhibited distinct peak expression patterns across all stages: 24 DEGs peaked in June, 8 in August, 12 in October, 17 in April, and 7 in December (**Figure 10A**). Specifically, *SsPAL*, *SsC4H*, *Ss4CL*, *SsC3’H*, *SsCAD*, *SsCoAOMT* (caffeoyl-CoA O-methyltransferase), *SsCOMT* (caffeic acid O-methyltransferase), *SsCSE*, and *SsF5H* showed high expression in June; *SsHCT* and *SsSCPL* (serine carboxypeptidase-like) peaked in October; *SsUGT72E* (UDP-glycosyltransferase 72E) in December; and *SsCCR* and *peroxidase* in April (**Figure 10A**). At the metabolic level, 8 DSMs were identified (**Figure 10A**), including 6 G/H-lignin intermediates: coniferyl alcohol, caffeate, *p*-coumaryl alcohol, *p*-coumaric acid, *p*-coumaraldehyde, and cinnamaldehyde. These metabolites displayed a fluctuating expression trend, with peak accumulation in August (**Figures 4A, B**, **10A**). Correlation analysis revealed significant positive correlations among the metabolites: coniferyl alcohol, *p*-coumaryl alcohol, and *p*-coumaraldehyde were positively correlated with *p*-coumaric acid or coniferyl alcohol, and *p*-coumaryl alcohol was also positively correlated with caffeate and *p*-coumaraldehyde (**Figure 10B**). At the gene–metabolite level, *Ss4CL* and *SsC4H* were positively correlated with sinapyl alcohol, while *SsUGT72E* showed a positive correlation with *p*-coumaraldehyde (**Figure 10B**).

To further investigate the regulation of the PB pathway, a regulatory network was constructed. The accumulation of coniferyl alcohol and *p*-coumaryl alcohol was positively regulated by several key genes, including *SsCOMT* (*DN23680_c0*), *SsCCoAOMT* (*DN18429_c2*), *peroxidase* (*DN14485_c0* and *DN17524_c0*), *SsHCT* (*DN6938_c0*, *DN23997_c3*, *DN12510_c0*, and *DN18090_c2*), *SsF5H* (*DN26124_c3*), *4CL* (*DN23189_c3*), *SsUGT72E* (*DN14539_c1*), *SsC3’H* (*DN28256_c0* and *DN19870_c2*), *SsPAL* (*DN19069_c3* and *DN19069_c1*), *SsC4H* (*DN22888_c1*), *SsMYB* (*DN18436_c0*), and *SsWOX* (*DN19350_c3*) (**Figure 10C**). This network highlights the coordinated interactions between key structural genes and metabolites within the PB pathway.

### 3.7 Integrated transcriptomic and metabolomic analyses revealed key regulatory nodes within the PHST pathways

**Figure 11:**
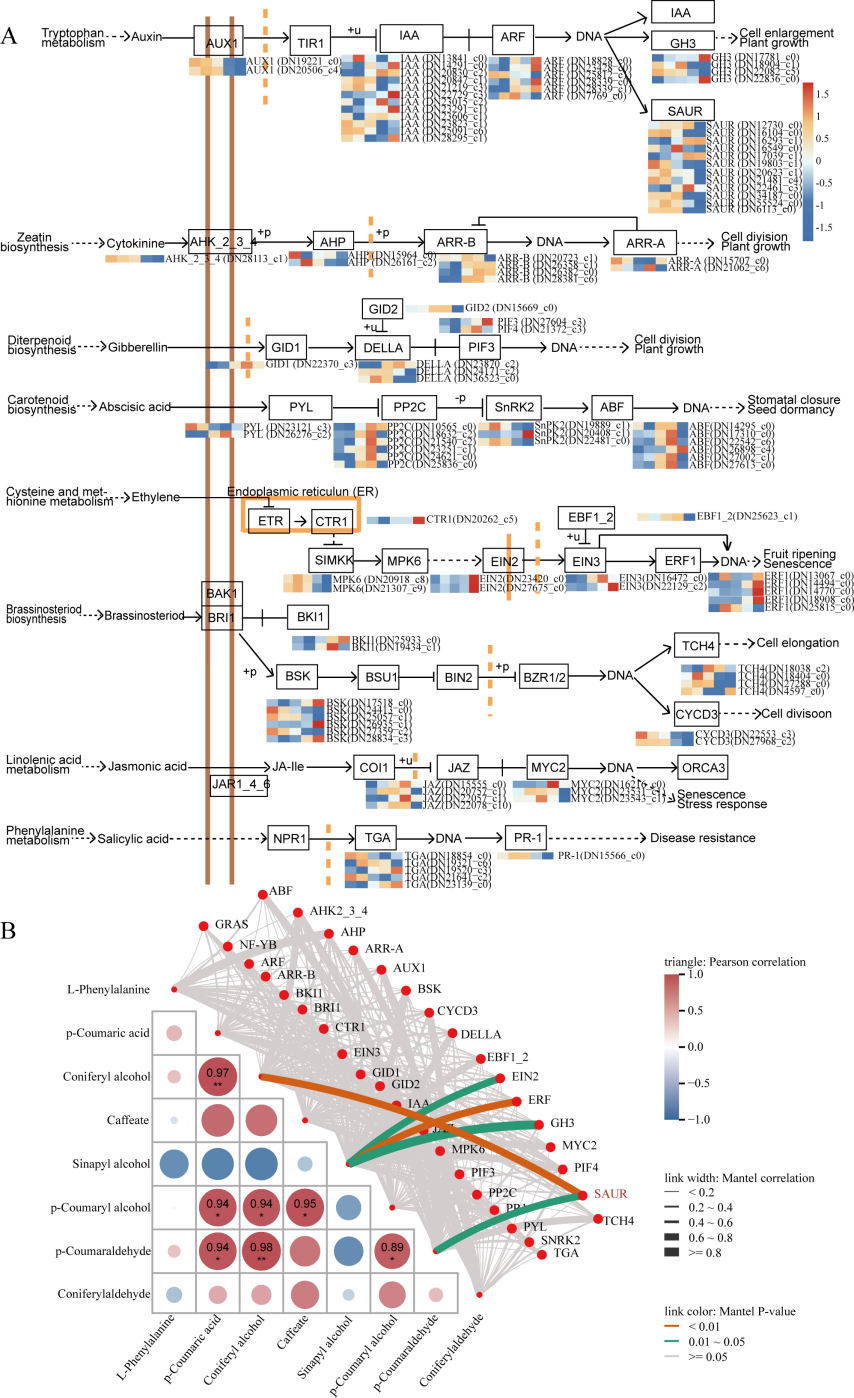
Analysis of DEGs and DSMs in the PHST pathway. **(A)** Expression analysis of DEGs in the PHST pathway. **(B)** Correlation analysis between PB-related DSMs and PHST-related DEGs. Lines in the lower-left indicate Mantel test results, with line thickness representing the overall correlation coefficient and line colour denoting significance. The triangular heatmap displays correlations among metabolites, with cell colours reflecting correlation strength (red: positive; blue: negative).

Across the seasonal cambial activity of *S. superba*, 109 PHST-related DEGs were identified, including 36 AUX, 17 ABA, 15 BR, 12 ETH, 9 CTK, 7 GA, 7 JA, and 6 SA genes (**Figure 11A**). In the AUX pathway, *SsAUX1* generally exhibited a downregulation trend, whereas *SsIAA* (Indole-3-Acetic Acid) and *SsGH3* (gretchen hagen 3) genes were predominantly upregulated (**Figure 11A**). *SsARF* and *SsSAUR* genes displayed an initial upregulation followed by downregulation, peaking in August (**Figure 11A**). Within the CTK pathway, *SsAHK2_3_4* (*Arabidopsis* histidine kinase 2/3/4), *AHP* (*Arabidopsis* histidine-containing phosphotransfer protein), and *SsARR-A* genes were largely downregulated, whereas *SsARR-B* followed by downregulation, peaking in October (**Figure 11A**). In the GA pathway, both *SsGID1* and *SsDELLA* genes showed a pattern of initial upregulation followed by downregulation, peaking in October and August, respectively, whereas *SsPIF3* (phytochrome-interacting factor 3) expression was continuously upregulated (**Figure 11A**). In the ETH pathway, *SsCTR1* (constitutive triple response 1), *SsEIN2* (ETH-insensitive 2), and *SsERF2* genes exhibited sustained upregulation (**Figure 11A**). For the ABA pathway, most related genes exhibited an initial upregulation followed by downregulation, with *SsPYL* (pyrabactin resistance 1-like), *SsPP2C*, and *SsABF* peaking in October (**Figure 11A**). Within the SA pathway, *SsTGA* was continuously upregulated (**Figure 11A**). In the JA pathway, *SsMYC2* and *SsJAZ* genes showed upregulation followed by downregulation, peaking in June and October, respectively (**Figure 11A**). Correlation analysis revealed significant positive correlations between *SsSAUR* and coniferyl alcohol, *p*-coumaraldehyde, whereas *SsGH3*, *SsERF*, and *SsEIN2* were positively correlated with sinapyl alcohol (**Figure 11B**). These results indicate a close regulatory relationship between plant hormone pathways and lignin biosynthesis.

### 3.8 qRT-PCR analysis

**Figure 12:**
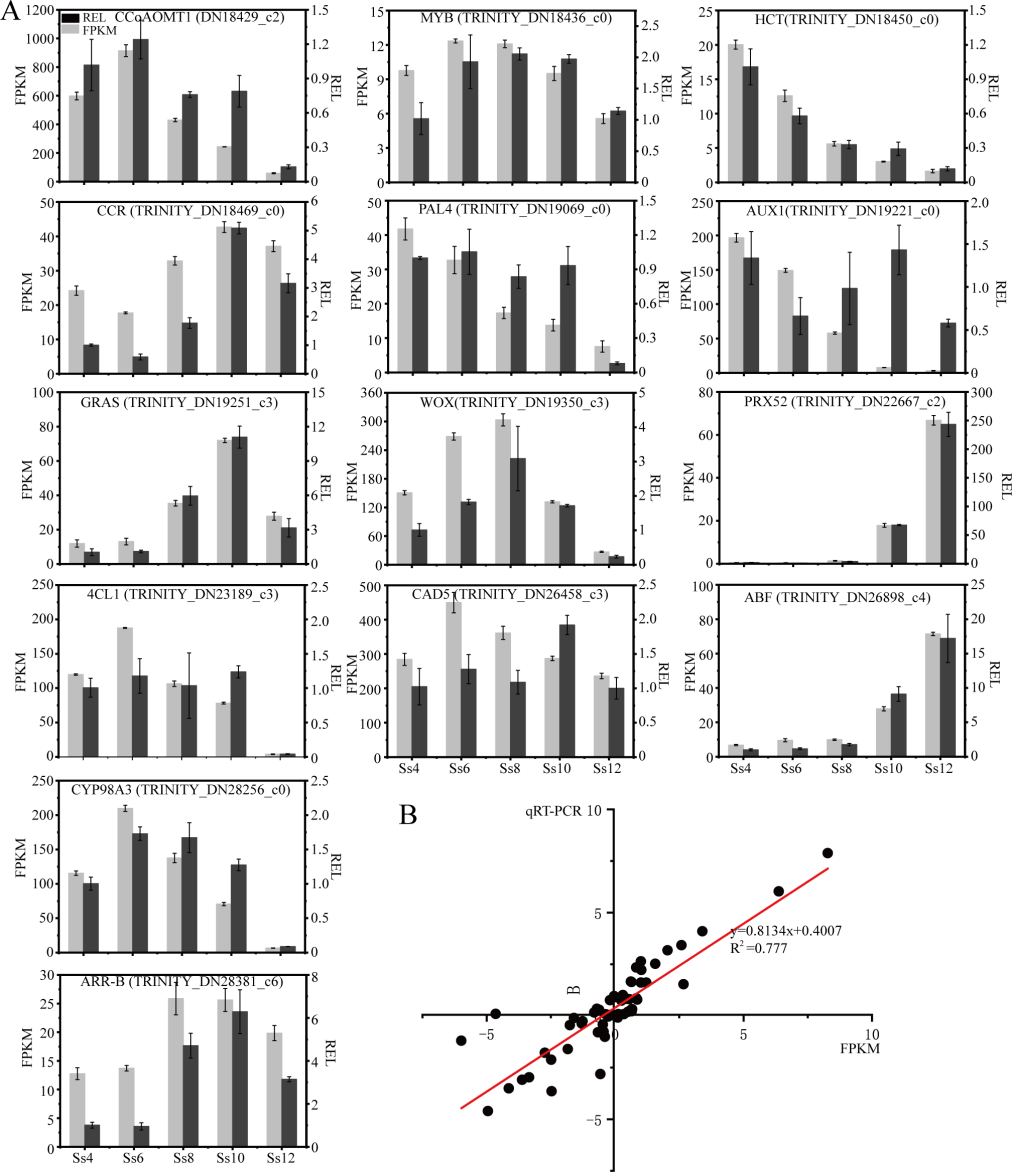
Expression of genes as determined by qRT-PCR. **(A)** Expression of genes as determined by qRT-PCR. **(B)** Linear fitting of qRT-PCR data and FPKM data were performed after normalizing their row data with Ss4 data as a reference, respectively. The *x*-axis represents FPKM data, and the *y*-axis represents qRT-PCR data.

To validate the reliability of the transcriptome data, qRT-PCR was performed on 14 genes involved in the PB and PHST pathways, as well as several TFs. The relative expression trends of 13 genes were consistent with the FPKM values obtained from RNA-seq (**Figure 12A**). Specifically, nine genes displayed an upregulation followed by a decline, two exhibited a continuous upward trend, and two showed a downward trend (**Figure 12A**). Overall, the qRT-PCR expression trends closely mirrored the RNA-seq data, with a correlation coefficient of R² = 0.777 (**Figure 12B**). These results confirm the reliability of the transcriptome data and provide a solid foundation for elucidating the metabolic regulatory network underlying cambial activity in *S. superba*.

## 4 Discussion

### 4.1 Dynamic regulation of the PB pathway and its conserved function in cambial activity

The PB pathway is a plant-specific secondary metabolic pathway that plays a central role in lignin biosynthesis, cell wall thickening, stress resistance, and overall plant growth and development (Behr et al., 2020; Liu et al., 2018; Yang et al., 2024a, b, c; Zhao and Dixon, 2011). In this study, we identified 8 PB-related DSMs, including various G- and H-type lignin precursors such as coniferyl alcohol, *p*-coumaryl alcohol, and their corresponding acids and aldehydes (**Figures 4A**, **10A**). These metabolites accumulated during the early to mid-stages of cambial active period (June and August), followed by a gradual decline, exhibiting a typical “upregulation followed by downregulation” pattern (**Figures 4A, B**, **10A**). suggesting that their functional peak coincides with active cambial proliferation and xylem differentiation. Notably, sinapyl alcohol, the primary precursor of S-type lignin, showed a continuous increase throughout the process (**Figures 4A, B**), indicating its sustained importance during the later stages of secondary wall thickening. This accumulation may enhance cell wall density and improve water transport efficiency in vessels, thereby strengthening xylem structure and function (Vanholme et al., 2010). Transcriptomic analysis revealed dynamic expression of key structural genes in the PB pathway, including *SsPAL*, *SsC4H*, *Ss4CL*, *SsHCT*, *SsC3’H*, *SsF5H*, *SsCCR*, and *SsCAD*, closely correlating with changes in the associated metabolites (**Figure 10A**). This “transcription–metabolism coupling” indicates that PB pathway activity is largely governed at the transcriptional level, enabling plants to respond rapidly to developmental cues or environmental stimuli (Zhao and Dixon, 2011). Previous studies in poplar (*Populus trichocarpa*) and *A. thaliana* have shown that the spatiotemporal expression of PB pathway genes is closely linked with vascular cambium activity, with key lignin biosynthesis genes highly expressed during active cambial phases (Zhang et al., 2024a, b). Similarly, in other woody plants, including eucalypt (*Eucalyptus grandis*) (Harakava, 2005), *C. fortunei* (Yang et al., 2024c; Zhang et al., 2022), *C. lanceolata* (Yang et al., 2023, 2025), and spruce (*Picea abies*) (Emiliani et al., 2011), the PB pathway remains highly active during peak secondary growth. These results underscore the conservation of the PB pathway across species, both in its functional role and regulatory patterns during seasonal cambial activity, underscoring its critical role as a core module in secondary growth.

This study identified a coexpression module significantly associated with the PB pathway, encompassing multiple key structural genes as well as regulatory factors such as *SsMYB*, *SsbHLH*, *SsWOX*, and *SsGRAS* (**Figures 9F, G**). These findings suggest that during cambial and secondary growth, the dynamic activation of the PB pathway may depend on the cooperative actions of diverse TFs, enabling precise spatial and temporal regulation of target gene expression (Zhao and Dixon, 2011). Among these TFs, *MYBs* play a particularly central role. Extensive evidence has demonstrated that *MYBs* are core regulators of lignin biosynthesis. They can directly bind to the promoter regions of key structural genes such as *PAL*, *C4H*, and *4CL*, thereby initiating or enhancing transcriptional activity and influencing lignin precursor synthesis and accumulation (Zhao and Dixon, 2011; Zhong et al., 2006). For instance, in *P. trichocarpa*, *PtrMYB3* and *PtrMYB20* activate several lignin biosynthesis genes, controlling secondary cell wall formation (McCarthy et al., 2009). In *A. thaliana*, *AtMYB46* and *AtMYB83* act as “master regulators,” with their overexpression significantly upregulating numerous cell wall biosynthesis genes (Zhong et al., 2006). Consistent with these findings, several *SsMYB* family members in *S. superba* were upregulated within PB-related modules and exhibited strong coexpression with structural genes (**Figure 9B**), supporting their pivotal role in PB pathway regulation. *WOX* TFs also emerged as important components of the PB-related module. The *WOX* family is widely involved in maintaining meristem activity, determining cell fate, and regulating organogenesis (van der Graaff et al., 2009). In *A. thaliana*, *AtWOX4* is highly expressed in the vascular cambium, interacting with the TMO5/LHW pathway to regulate cambial stem cell proliferation (Petzold et al., 2018; Suer et al., 2011; Zhou et al., 2024). Similarly, the *SsWOX* members detected in *S. superba* may contribute to cell fate determination and differentiation across cambial regions, offering insights into the spatial regulatory mechanisms of vascular tissues in woody plants. In summary, the PB pathway in *S. superba* exhibits pronounced dynamic regulation throughout the seasonal cambial activity stages. Structural genes and DSMs are tightly synchronized across these stages, highlighting the PB pathway as a conserved functional module in secondary growth. Its evolutionary conservation and consistent regulatory patterns across species suggest that it is governed by a finely tuned transcriptional network that modulates cambial activity.

### 4.2 Multilevel synergistic mechanisms of PHST in the spatiotemporal regulation of cambial activity

Phytohormones are essential signalling molecules that regulate plant growth and development, and responses to environmental stimuli. They are central to vascular tissue development, particularly in the initiation, maintenance, and differentiation of the cambium (Ding et al., 2024; Wang, 2020). Through the establishment of spatial gradients, modulation of polar transport, and activation of downstream gene expression, phytohormones generate a complex, hierarchical, and dynamic regulatory network. This coordinated system ensures proper cambial function, directing cell division and differentiation, and modulating vascular tissue formation in response to both seasonal cambial activity and environmental signals.

Auxin plays a pivotal role in regulating cambial stem cell activity, particularly by modulating the expression of AUX-related factors essential for the transition between dormant and active cambial states (Hayashi, 2012; Nilsson et al., 2008). During the active cambial phase (June–August), genes associated with AUX biosynthesis and signalling, including *SsARF*, *SsAUX1*, and *SsSAUR*, were upregulated (**Figure 11A**). Through polar transport mediated by *PIN* and *AUX1*, AUX establishes concentration gradients within the cambium, particularly towards the xylem side, thereby directing cambial cells to differentiate into xylem tissue. This gradient-dependent regulatory mechanism has been demonstrated in model plants such as *A. thaliana* and *P. trichocarpa* (Immanen et al., 2016; Uggla et al., 2001). Our findings confirm that this mechanism is highly conserved and adaptive in perennial woody species, underscoring the central role of IAA polar transport and signalling in maintaining and differentiating the vascular cambium. Within the signalling pathway, *ARFs* acts as key TFs by binding to AuxRE elements in the promoters of downstream genes (Cherenkov et al., 2017), thereby activating or repressing transcription programmes involved in cell division, elongation, and differentiation (Brackmann et al., 2018; Hu et al., 2022; Mao et al., 2019). In *S. superba*, several *SsARF* genes were upregulated during the active cambial phase (**Figure 11A**), highlighting their crucial role in cell fate determination and vascular activity (Brackmann et al., 2018; Hu et al., 2022). Moreover, the *SAUR* gene family, as early responders to AUX signalling, is closely associated with cell elongation. Previous studies in *A. thaliana*, maize (*Zea mays*), and *Populus* have shown that *SAUR* genes promote the expression of cell wall-loosening factors such as *PME* and *EXP*, thereby facilitating cell extension (Spartz et al., 2014). Consistently, in our study several *SsSAUR* genes were enriched during the active cambial period (**Figure 11A**), further supporting their role in promoting cambial cell elongation. These findings underscore the pivotal role of AUX in coordinating cambial activity, thereby establishing a finely tuned regulatory network underlying secondary growth in woody plants.

In addition to AUX, GAs and CTKs are crucial hormonal signals involved in seasonal cambial activity, acting synergistically within the multilayered regulatory network. GA primarily regulates cell cycle progression and cell expansion, functioning in concert with AUX to coordinate the proliferation and differentiation of cambial cells. In *Populus* and *A. thalianas*, GA binds to its receptor GID1, which subsequently induces the degradation of the growth-repressing DELLA proteins. DELLA proteins, as negative regulators of GA signalling, inhibit cell growth and differentiation under stable conditions. Their degradation releases this inhibition, thereby promoting cell proliferation and secondary cell wall thickening, enhancing xylem cell function and facilitating secondary growth (Mauriat and Moritz, 2009). In this study, during the active cambial phase (August–October), genes involved in GA biosynthesis and signalling, including the *SsGID1* receptor and *SsDELLA*-coding genes, were upregulated (**Figure 11A**), highlighting the pivotal role of GA signalling in regulating cambial cell cycle progression and secondary wall formation. GA acts in concert with AUX signalling to drive the directional and functional differentiation of cambial cells towards xylem tissue. In contrast, CTK primarily regulates the division and maintenance of cambial cells, thereby ensuring the continuous activity of the vascular cambium. CTK signalling, through regulation of cell cycle-related genes, sustains the proliferative potential of cambial cells and complements AUX signalling, achieving a dynamic balance between cell division and differentiation. In *A. thalianas*, CTK regulates WOX TFs to maintain stem cell populations, thereby ensuring the self-renewal capacity of the cambium (Müller and Sheen, 2008). Consistently, transcriptome data of *S. superba* cambium revealed significant temporal expression patterns of CTK biosynthesis and signalling response genes, particularly the *SsARR-Bs* (**Figure 11A**), further underscoring their crucial role in maintaining cambial activity.

In the complex regulatory network underlying seasonal cambial activity, key TFs such as *SsGRAS*, *SsSAUR*, and *SsNF-YB* not only integrate multiple phytohormone signals (**Figure 9G**) but also closely interact with the PB pathway, jointly regulating cell fate determination and lignification. Increasing evidence suggests that PB pathway activity is finely controlled by TFs and is further modulated through integration with phytohormone networks (Chezem and Clay, 2016). The *GRAS* family, acting as GA-responsive TFs, can indirectly influence the expression of PB pathway genes by regulating key GA signalling components, including DELLA proteins (Luo et al., 2024; Yang et al., 2021). GA signalling promotes the upregulation of genes such as *PAL*, *4CL*, and *CCR*, thereby accelerating lignin synthesis and deposition (Mauriat and Moritz, 2009). In this study, *SsGRASs* were coexpressed with key PB pathway enzymes (**Figure 9G**), suggesting that they may function as a bridge between GA signalling and the PB pathway, orchestrating lignification. The *SAUR* gene family, as early-response elements in AUX signalling, plays a central role in cell elongation, cell wall remodelling, and wall loosening (Kathare et al., 2018; van Mourik et al., 2017). SAUR proteins activate the plasma membrane H^+-ATPase to facilitate cell wall loosening, indirectly influencing the expression of PB pathway structural genes. For example, in *Populus* and *A. thaliana*, *SAUR* expression levels are positively correlated with PB pathway activity, highlighting the potential role of IAA signalling via *SAUR* in regulating lignin biosynthesis (Yun et al., 2022). In this study, several *SsSAUR* genes showed high expression during cambial active period, with spatiotemporal dynamics closely aligned with key lignin biosynthesis enzyme genes (**Figure 9G**), further supporting their role in coordinating cell elongation and lignification. *NF-YB* TFs function as integrators of multiple hormonal signals in seasonal cambial activity. They are involved in cell cycle and stem cell maintenance, and can directly or indirectly regulate PB pathway gene expression, thereby influencing lignin synthesis (Petroni et al., 2012). In this study, *SsNF-YB* genes exhibited strong coexpression with CTK and IAA signalling pathway genes (**Figure 9G**) and showed significant correlation with key PB pathway genes, coordinating hormonal networks and metabolic pathways to regulate cell fate and lignification. Overall, *SsGRAS*, *SsSAUR*, and *SsNF-YB* act as core TFs at the intersection of multi-hormone signalling and the PB pathway in the vascular cambium. They form an integrated regulatory network that ensures the precise differentiation and functional maturation of cambial cells. By coordinating hormonal inputs and metabolic gene expression, these TFs provide a molecular foundation for secondary growth and environmental adaptation in woody plants. Future functional validation and mechanistic studies of these TFs will provide valuable insights and potential applications for improving wood quality and enhancing plant stress resilience.

## 5 Conclusions

This study integrated metabolomics and transcriptomics data, identifying 2,178 DSMs, 8 lignin-related DSMs, and 19,609 DEGs. Notably, the PB and PHST pathways were significantly enriched during the active cambial phase. WGCNA further revealed key regulatory modules and core TFs, such as *SsMYB*, *SsbHLH*, *SsWOX*, *SsGRAS*, *SsSAU*R, and *SsNF-YB*. This study not only deepens our understanding of the regulatory mechanisms of vascular cambium activity in *S. superba*, but also provides valuable insights into the molecular regulation of vascular cambium activity, secondary growth, and lignin accumulation in woody plants. These findings contribute to molecular breeding and wood quality improvement in woody species.

## Data Availability

The datasets presented in this study are deposited in the NCBI BioProject repository, accession number PRJNA1312306.
