# Supplementary material for: Integrated transcriptomic, untargeted and targeted metabolomic analyses reveal seasonal regulatory mechanisms of vascular cambium activity in woody plants: insights from Schima superba
Source: Front Plant Sci. 2026 Jan 22;16:1727826. doi: 10.3389/fpls.2025.1727826 (PMC12872755; doi:10.3389/fpls.2025.1727826)
Supplement: Supplementary file 2 [file SupplementaryFile1.docx]

Supplementary Material

## Supplementary Figures

**
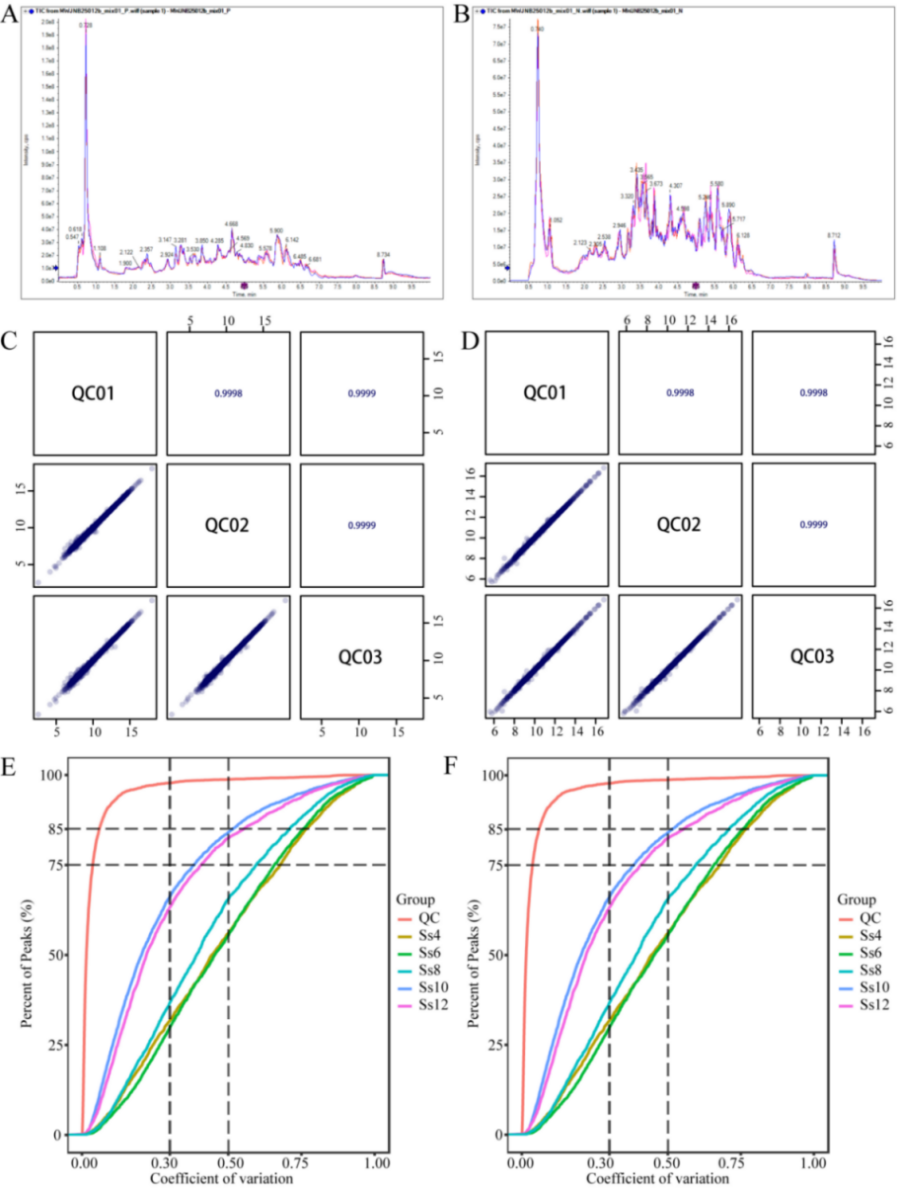
Supplementary Figure 1.** Sample quality control (QC) analysis. (A) Overlay of total ion chromatograms (TICs) for QC samples in positive ion mode. (B) Overlay of TICs for QC samples in negative ion mode. (C) Correlation plot of QC samples in positive ion mode. (D) Correlation plot of QC samples in negative ion mode. Diagonal cells indicate QC sample names; cells in the lower-left triangle show scatter plots of log-transformed metabolite abundances, with each point representing a single metabolite. Cells in the upper-right triangle display Pearson correlation coefficients between the corresponding QC samples. (E) Distribution of coefficients of variation (CVs) for samples in positive ion mode. (F) Distribution of CVs for samples in negative ion mode. The x-axis represents CV values, and the y-axis shows the proportion of metabolites with CVs below the corresponding value. Different colours denote different sample groups, with QC representing quality control samples. Vertical reference lines indicate CVs of 0.3 and 0.5, and horizontal reference lines correspond to proportions of 75% and 85% of total metabolites.


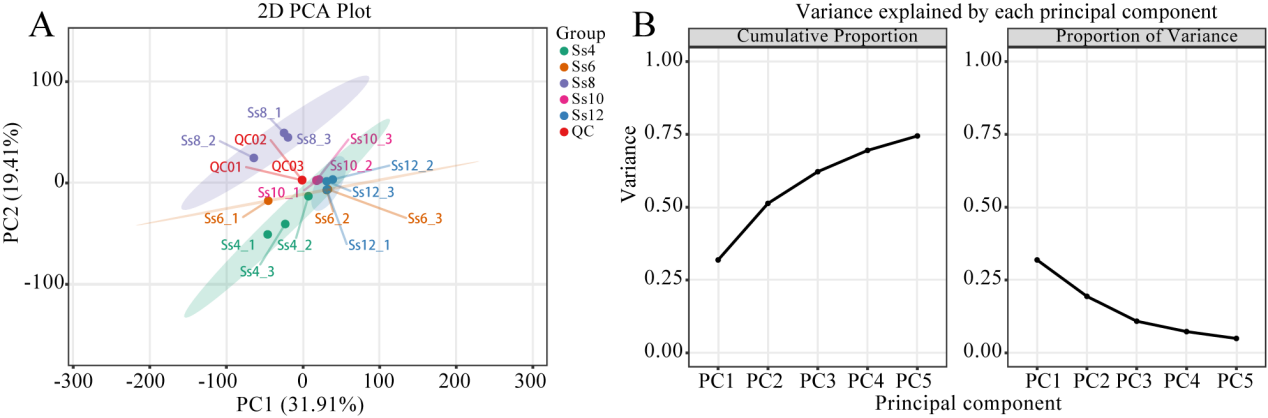


**Supplementary Figure 2.** Overall sample principal component analysis (PCA). (A) PCA score plot of all sample groups and QC samples based on metabolomic data. PC1, PC2, and PC3 represent the first, second, and third principal components, respectively, with percentages indicating the proportion of variance explained by each component. Each point represents a sample, and samples from the same group are shown in the same colour. Group indicates sample grouping. (B) Explained variance of principal components for each group. The *x*-axis represents the principal components, and the *y*-axis indicates the proportion of variance explained. The left panel shows the cumulative variance explained, and the right panel shows the variance explained by each individual principal component.

**Supplementary Figure
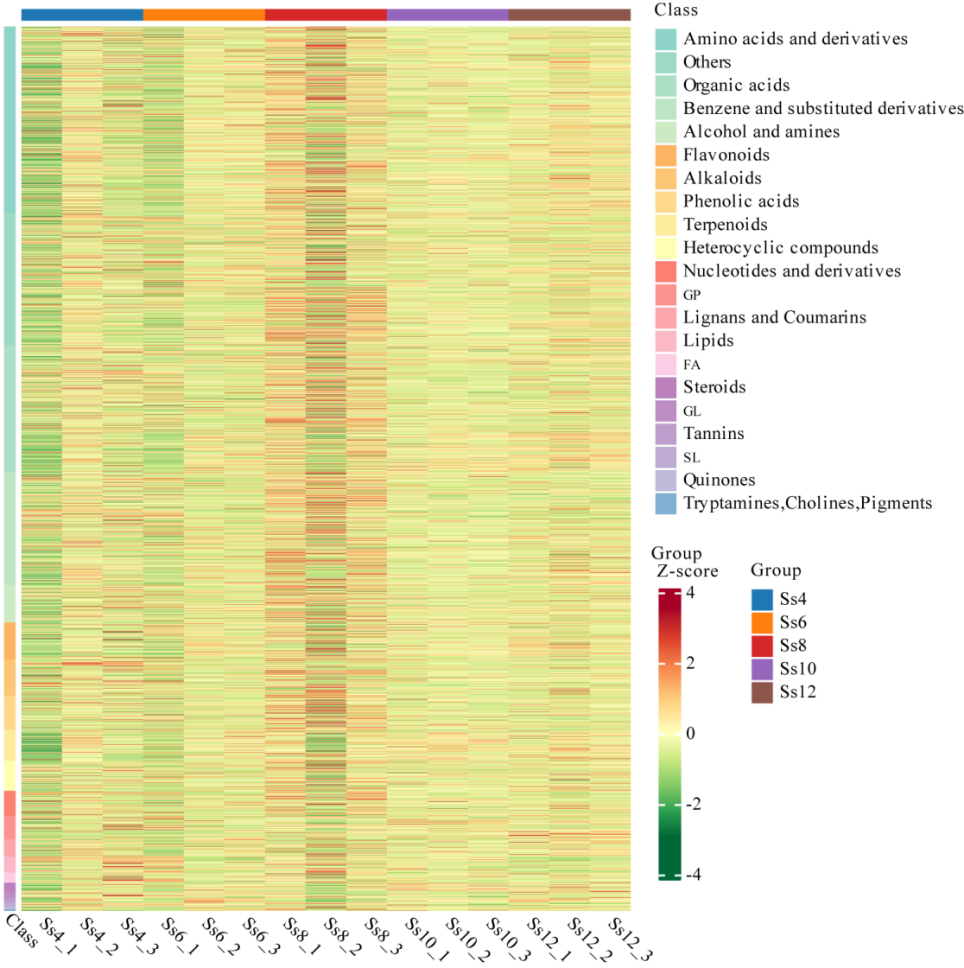
 3.** Overall clustering of samples. The *x*-axis represents sample names, and the *y*-axis represents metabolites. Group indicates sample grouping. Colours represent relative metabolite abundance after standardisation (red: high; green: low). All_heatmap_class: heatmap grouped by metabolite classification, with Class indicating the primary metabolite category. All_heatmap_col-row_cluster: hierarchical clustering of both metabolites and samples; dendrograms on the left show metabolite clustering, and dendrograms on the top show sample clustering. All_heatmap_row_cluster: hierarchical clustering of metabolites only, with dendrograms on the left indicating metabolite clustering.

**Supplementary Figure**
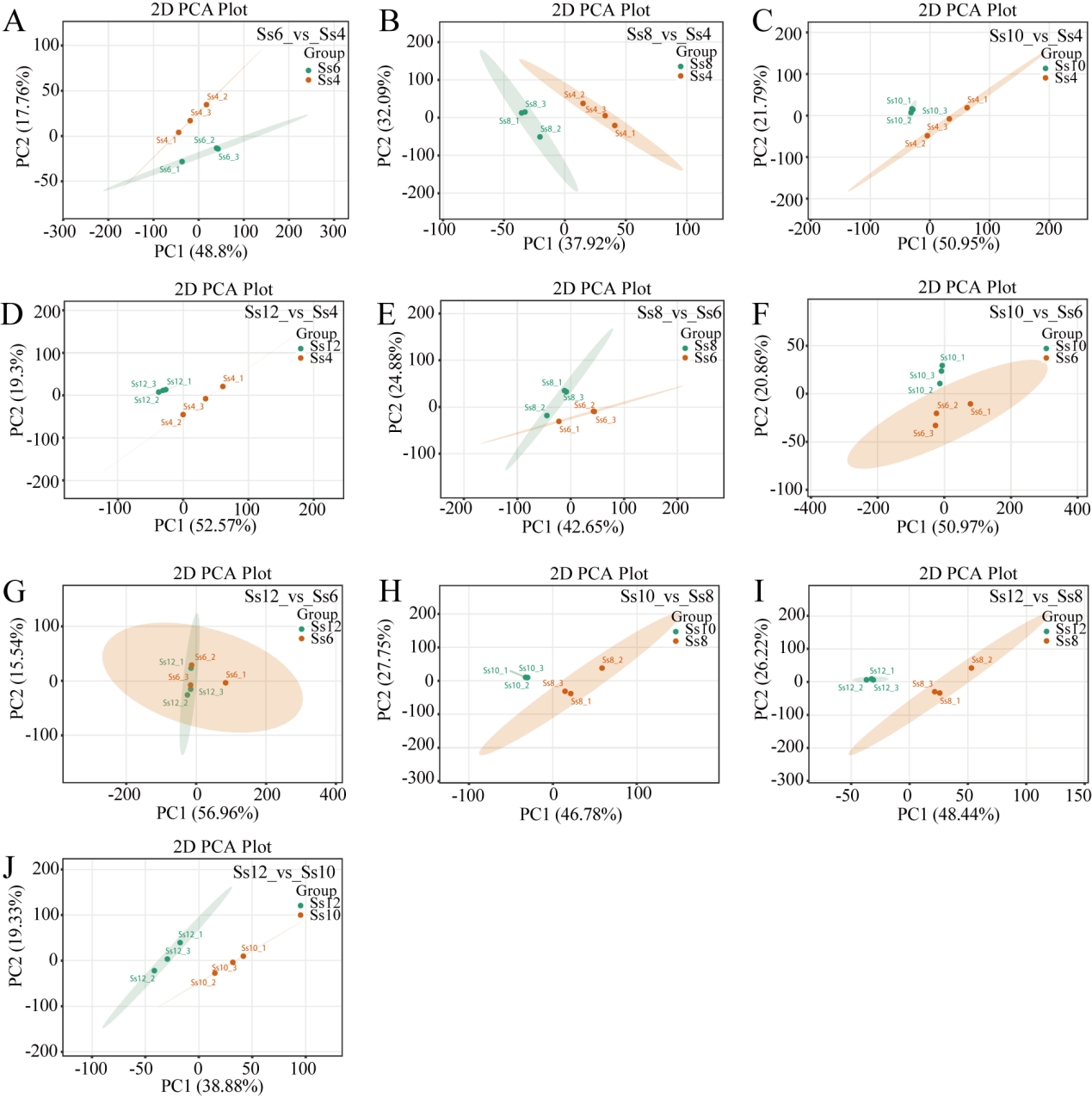
 **4.** PCA of grouped samples. (A) Ss6_vs_Ss4; (B) Ss8_vs_Ss4; (C) Ss10_vs_Ss4; (D) Ss12_vs_Ss4; (E) Ss8_vs_Ss6; (F) Ss10_vs_Ss6; (G) Ss12_vs_Ss6; (H) Ss10_vs_Ss8; (I) Ss12_vs_Ss8; and (J) Ss12_vs_Ss10. PC1 and PC2 represent the first and second principal components, respectively, with percentages indicating the proportion of variance explained by each component. Each point represents a sample, and samples from the same group are shown in the same colour. Group indicates sample grouping.


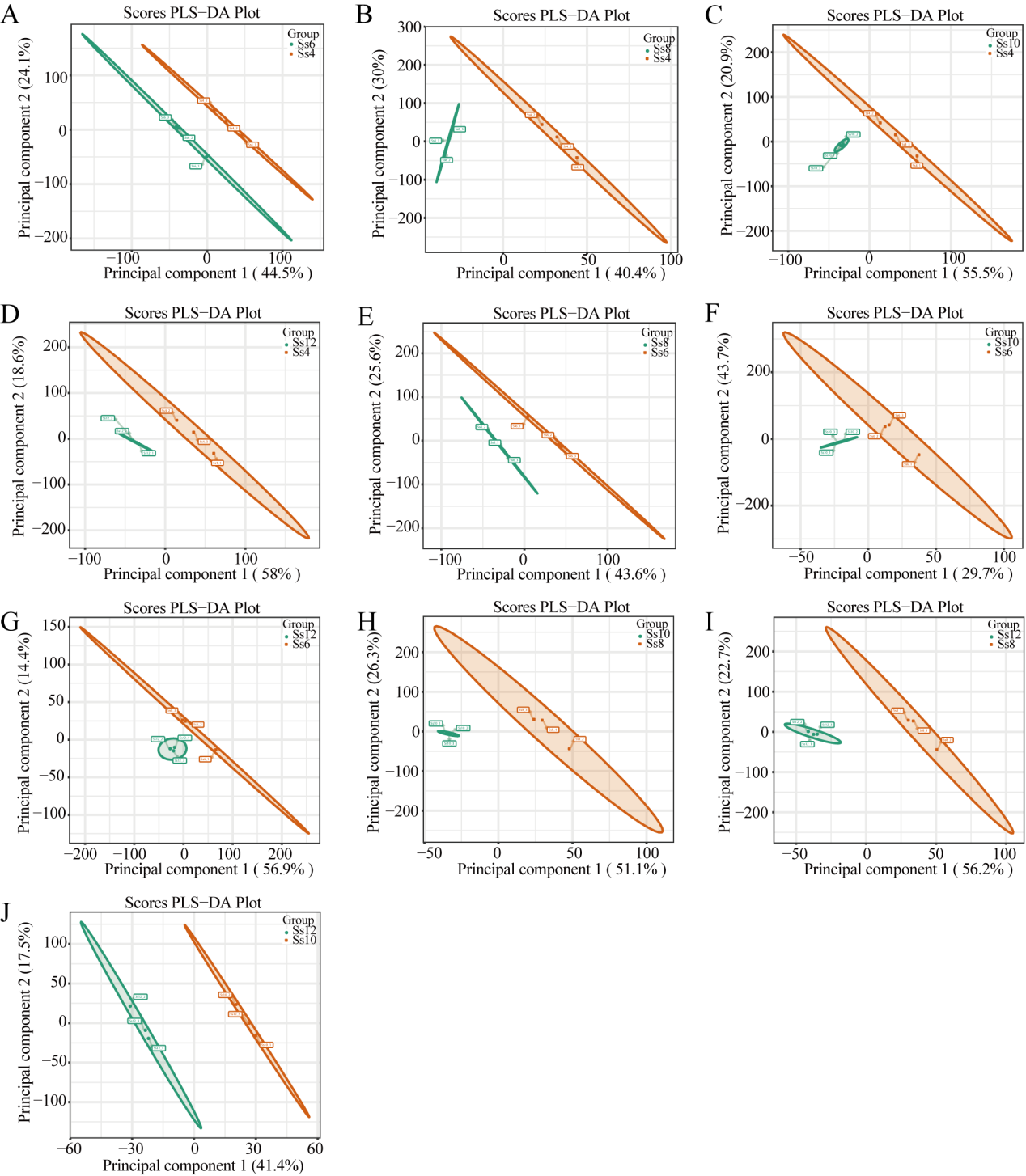


**Supplementary Figure 5.** PLS-DA of samples. (A) Ss6_vs_Ss4; (B) Ss8_vs_Ss4; (C) Ss10_vs_Ss4; (D) Ss12_vs_Ss4; (E) Ss8_vs_Ss6; (F) Ss10_vs_Ss6; (G) Ss12_vs_Ss6; (H) Ss10_vs_Ss8; (I) Ss12_vs_Ss8; and (J) Ss12_vs_Ss10. PC1 and PC2 represent the first and second principal components, respectively, with percentages indicating the proportion of variance explained by each component. Each point represents a sample, and samples from the same group are shown in the same colour. Group indicates sample grouping.


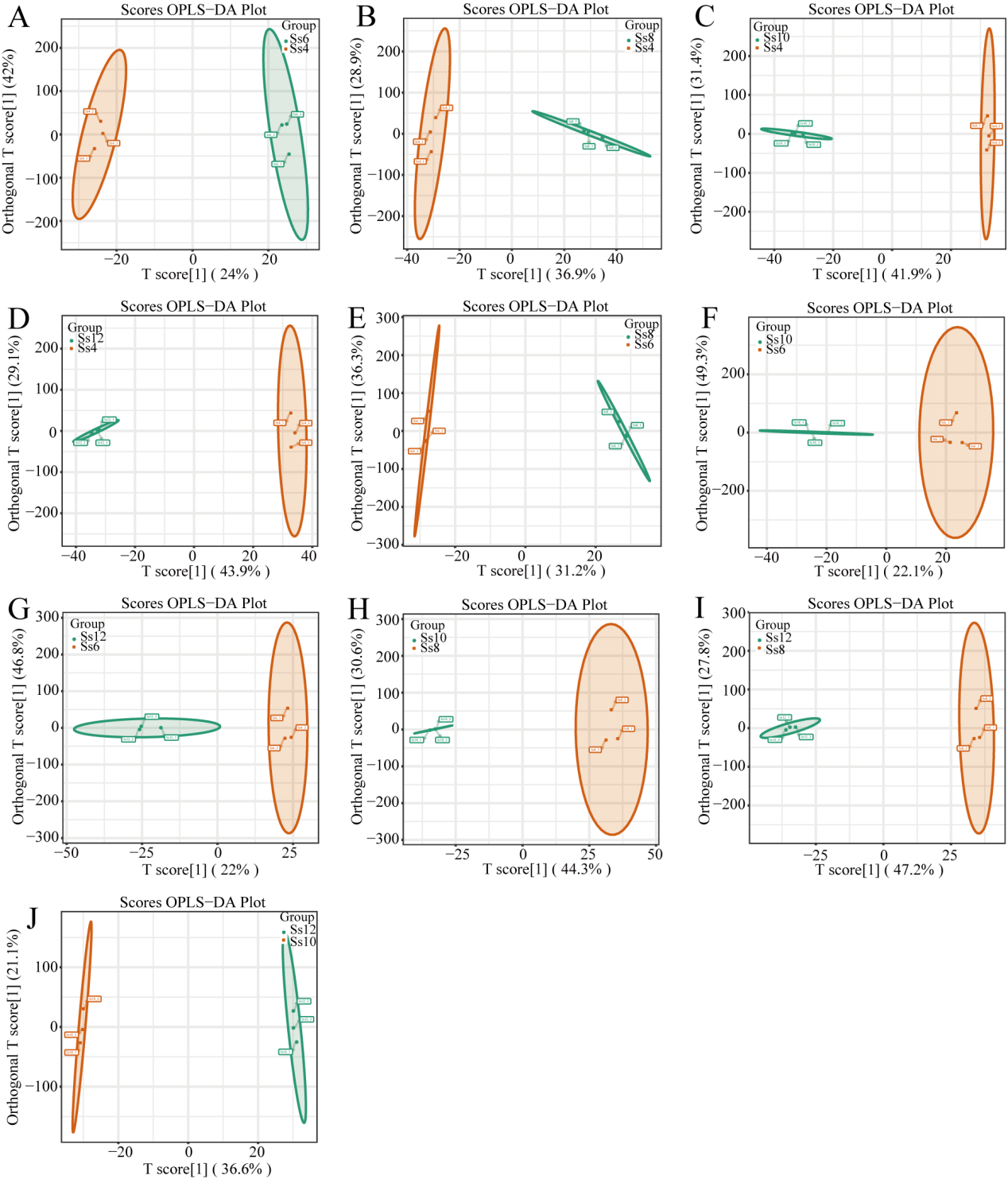
**Supplementary Figure 6.** OPLS-DA score plots. (A) Ss6_vs_Ss4; (B) Ss8_vs_Ss4; (C) Ss10_vs_Ss4; (D) Ss12_vs_Ss4; (E) Ss8_vs_Ss6; (F) Ss10_vs_Ss6; (G) Ss12_vs_Ss6; (H) Ss10_vs_Ss8; (I) Ss12_vs_Ss8; and (J) Ss12_vs_Ss10. The *x*-axis represents predictive components, which reflect differences between groups, and the *y*-axis represents orthogonal components, which reflect within-group variation. Percentages indicate the proportion of variance explained by each component. Each point represents a sample, and samples from the same group are shown in the same colour. Group indicates sample grouping.

**Supplementary Figure**
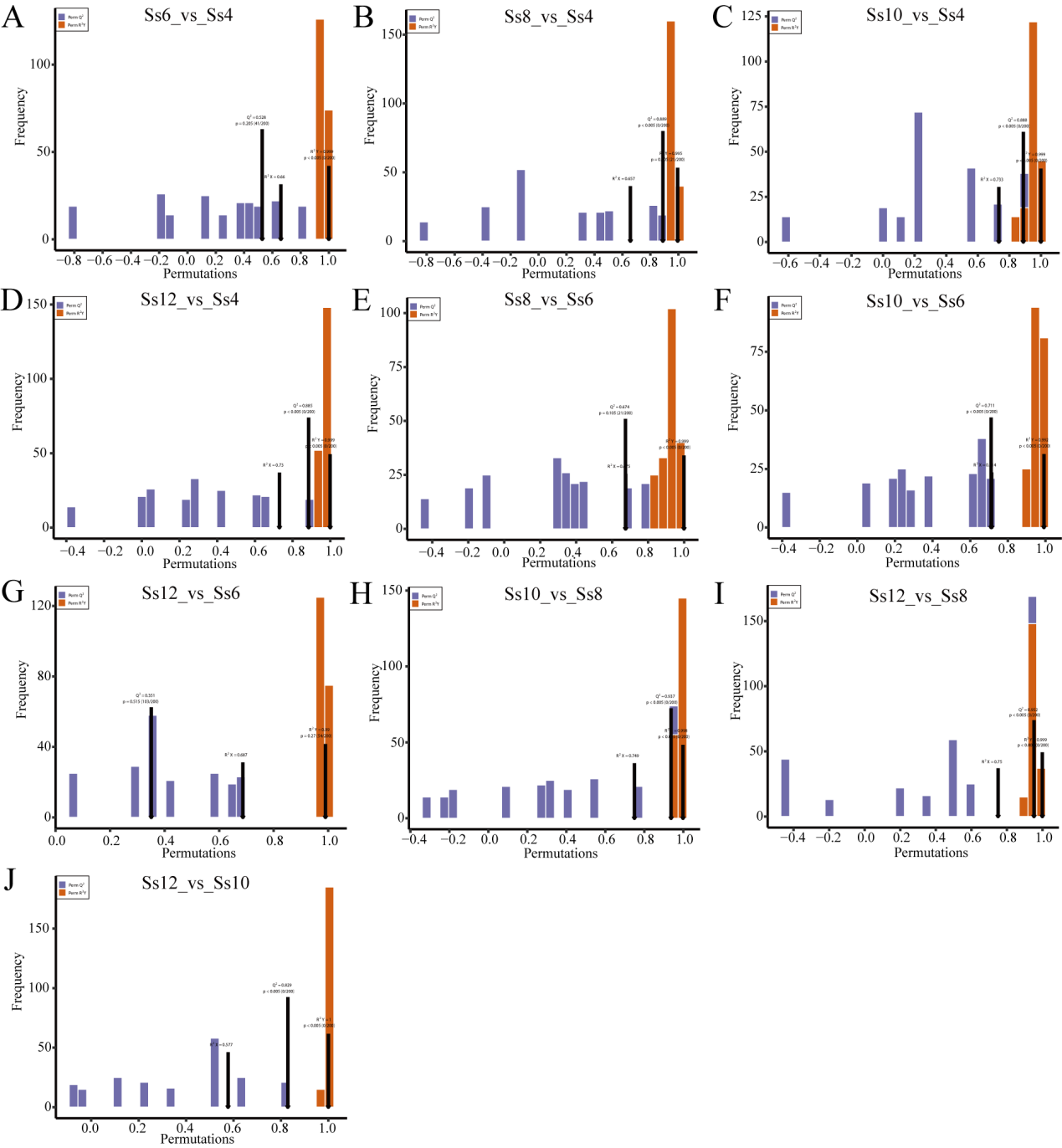
 **7.** OPLS-DA validation plots. (A) Ss6_vs_Ss4; (B) Ss8_vs_Ss4; (C) Ss10_vs_Ss4; (D) Ss12_vs_Ss4; (E) Ss8_vs_Ss6; (F) Ss10_vs_Ss6; (G) Ss12_vs_Ss6; (H) Ss10_vs_Ss8; (I) Ss12_vs_Ss8; and (J) Ss12_vs_Ss10. The x-axis represents the R²Y and Q² values of the model, and the y-axis indicates the frequency of classification results obtained from 200 random permutation tests. Orange bars represent R²Y values from randomly permuted models, purple bars represent Q² values from randomly permuted models, and black arrows indicate the R²X, R²Y, and Q² values of the original model.


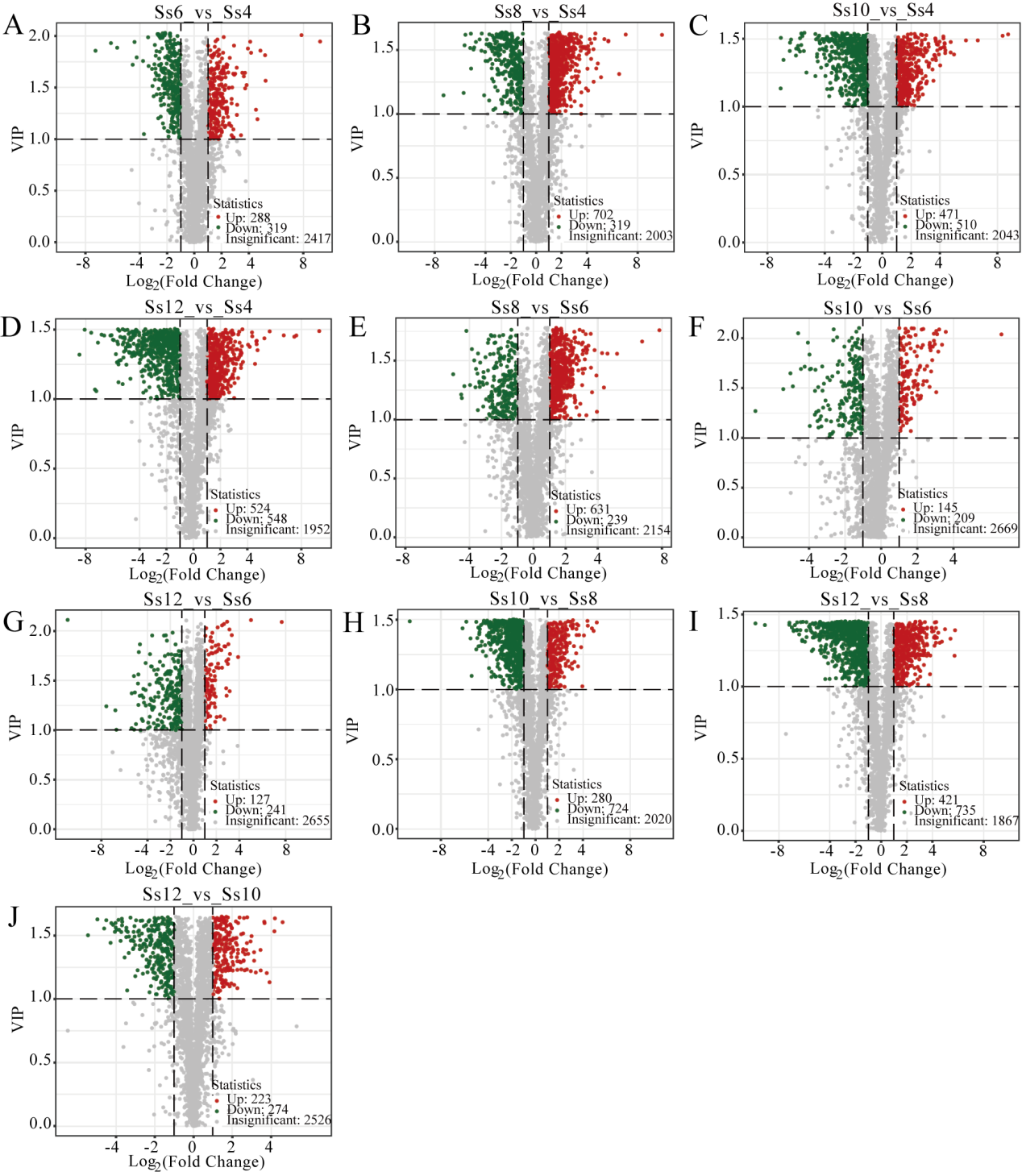


**Supplementary Figure 8.** Volcano plots of differential metabolites. (A) Ss6_vs_Ss4; (B) Ss8_vs_Ss4; (C) Ss10_vs_Ss4; (D) Ss12_vs_Ss4; (E) Ss8_vs_Ss6; (F) Ss10_vs_Ss6; (G) Ss12_vs_Ss6; (H) Ss10_vs_Ss8; (I) Ss12_vs_Ss8; and (J) Ss12_vs_Ss10. Each point in the volcano plot represents a metabolite. Green points indicate downregulated differential metabolites, red points indicate upregulated differential metabolites, and grey points represent detected metabolites that are not significantly different. The x-axis represents the log₂ fold change (log2FC) of relative metabolite abundance between the two sample groups; larger absolute values indicate greater differences between groups. Under the VIP + FC + P-value filtering criteria, the y-axis represents significance level (-log10P-value), and the size of each point corresponds to the VIP value. Under the VIP + FC filtering criteria, the y-axis represents the VIP value; higher values indicate more significant differences, with the identified differential metabolites considered more reliable.


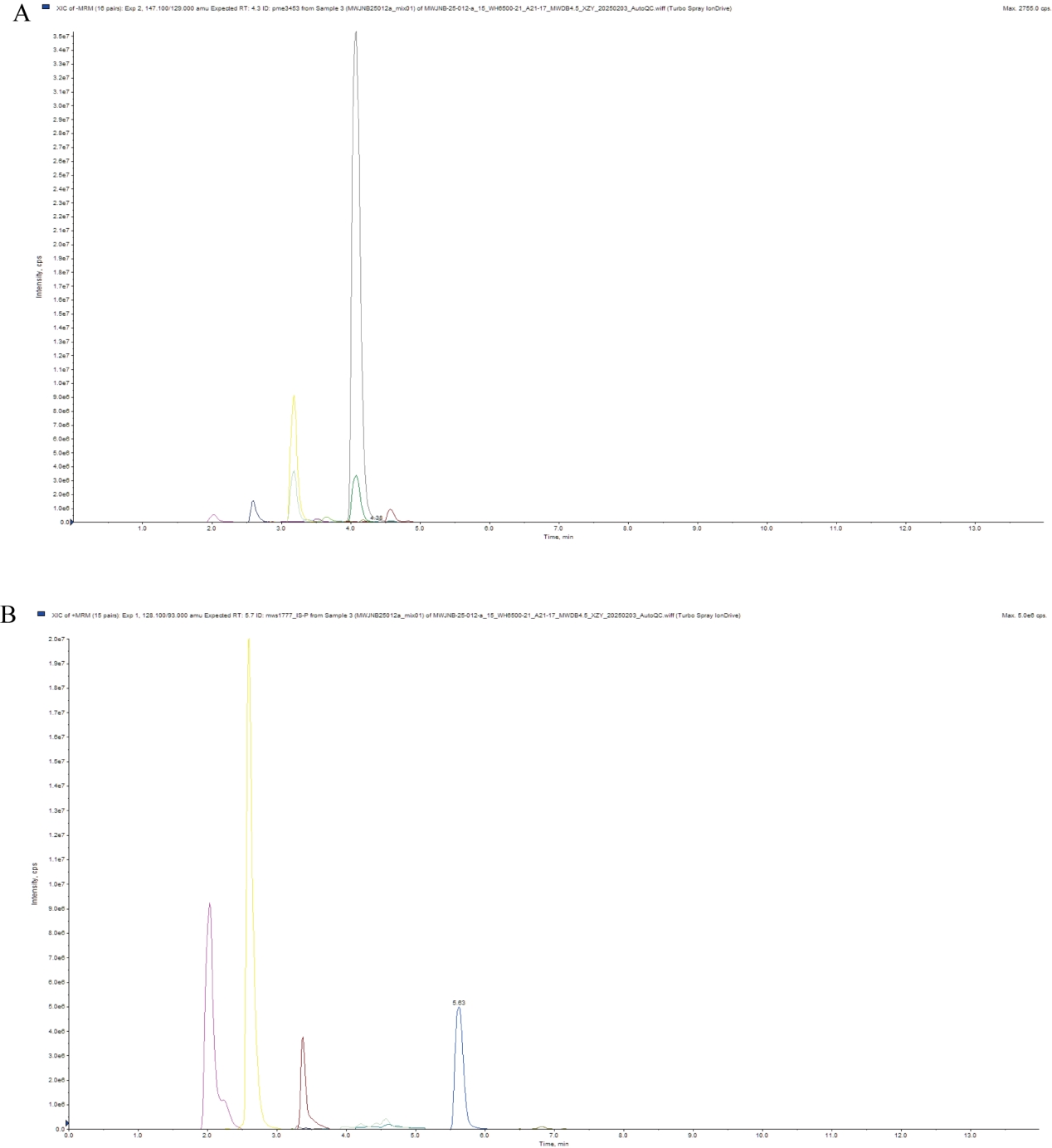
**Supplementary Figure 9.** MRM chromatograms of metabolites in samples. (A) Negative ion mode; (B) Positive ion mode. The x-axis represents the retention time (Rt) of detected metabolites, and the y-axis represents ion intensity (counts per second, cps).


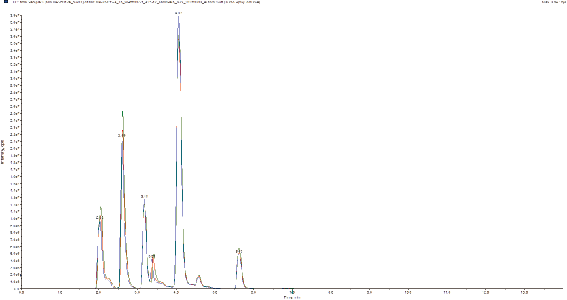


**Supplementary Figure 10.** Sample quality control analysis. Overlay of total ion chromatograms (TICs) for QC samples. The TIC overlay shows a high degree of overlap in both retention times and peak intensities, indicating that the mass spectrometer produced consistent signals for the same sample across different runs. This high instrument stability ensures the repeatability and reliability of the metabolomic data.


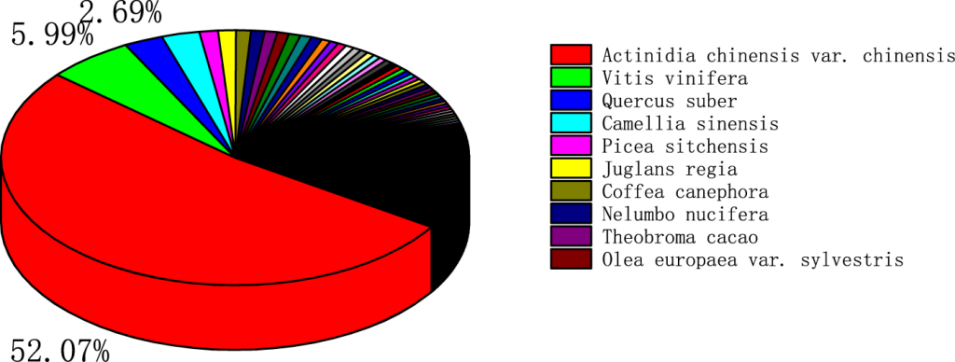


**Supplementary Figure 11.** NR annotation.


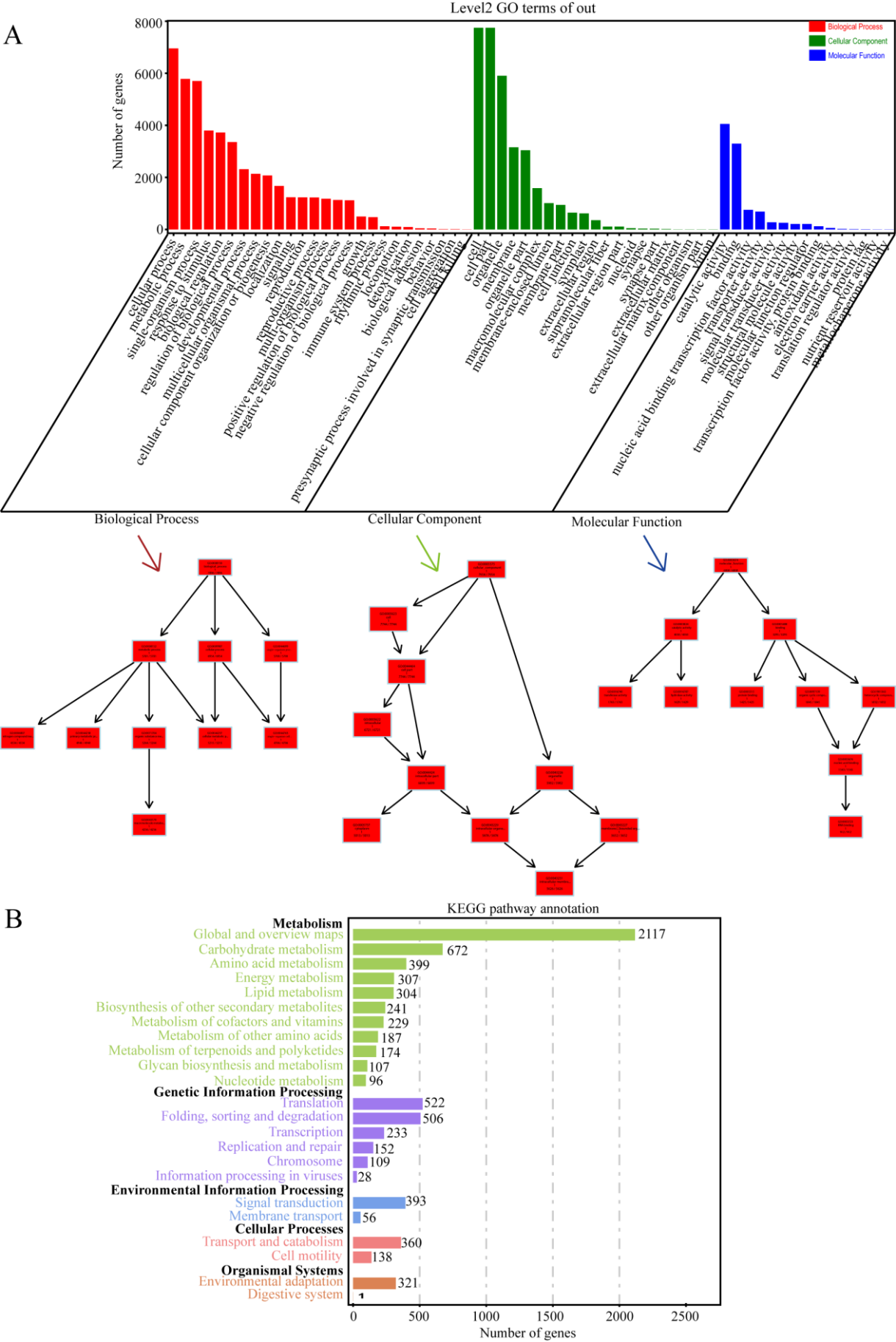
**Fig. S12.** GO and KEGG annotation. (A) Gene ontology (GO) annotation, classifying genes into biological processes, molecular functions, and cellular components. (B) Kyoto encyclopedia of genes and genomes (KEGG) annotation, categorising genes into metabolic and signalling pathways to infer their potential biological roles.


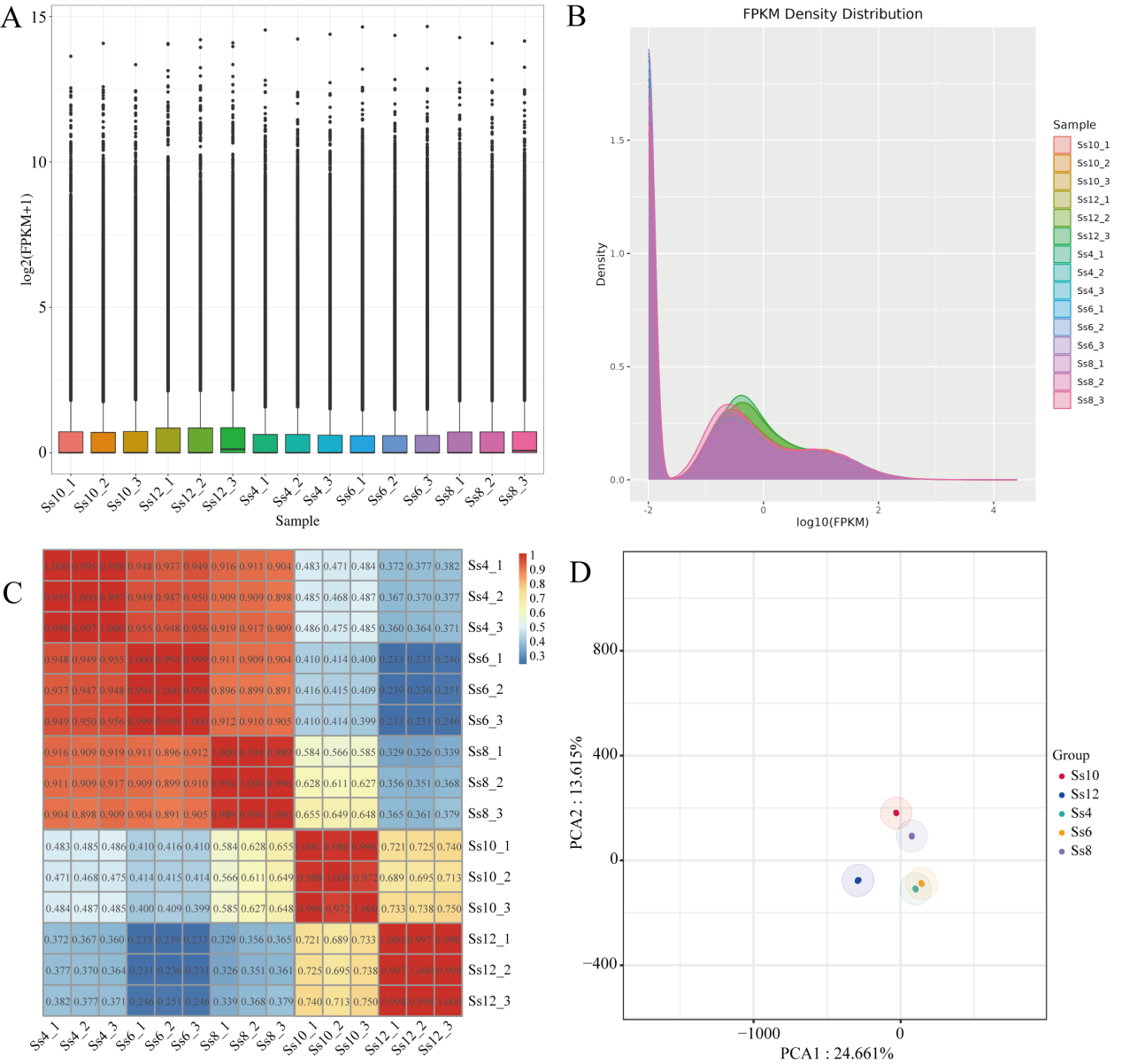


**Fig. S13.** Sample quality control. (A) Boxplot of FPKM values for all samples. (B) Coverage plot of FPKM values for all samples. (C) Heatmap of gene expression across samples. (D) Principal component analysis (PCA) plot of all samples.


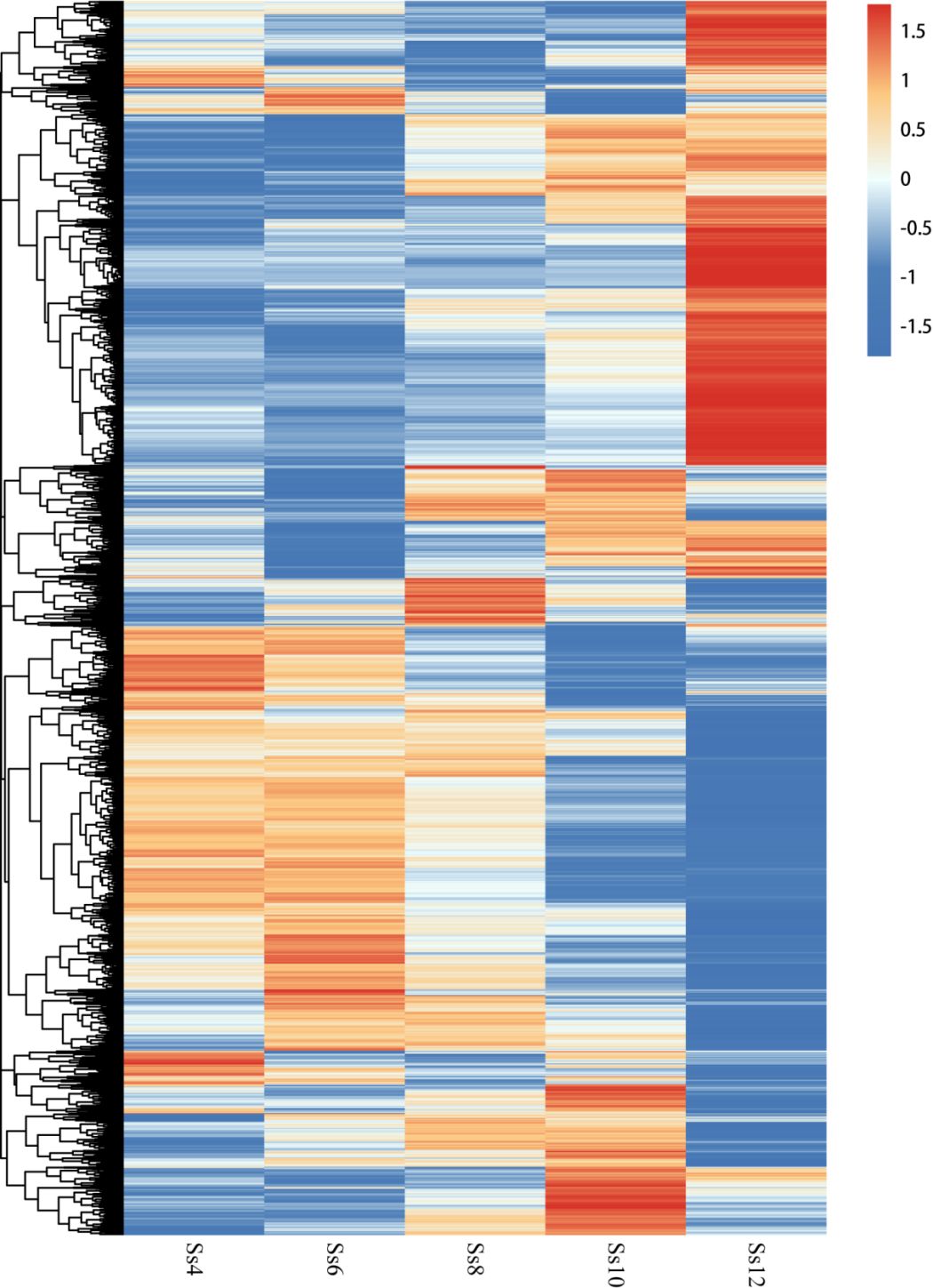


**Fig. S14.** Hierarchical clustering analysis (HCA) of all genes.

## Supplementary Tables

**Table S1.** Primer sequences used in this study.

| Gene | Forward primer sequence | Reverse primer sequence |
| --- | --- | --- |
| *TRINITY_DN18436_c0* | AACTGGTAAAAAGGGCGGC | GAAATAATCTGTGCTCATCCTCTGT |
| *TRINITY_DN19251_c3* | ACACTGCCCCATTTCTACCAC | CGTTCAACCTGTCCTGGCTATC |
| *TRINITY_DN19350_c3* | GGAAGCAGCAGGTTACAGGAC | CCACTTTTTTGGACTGTGAATCTAC |
| *TRINITY_DN23189_c3* | GAAGAGGCTGTTTCGGGGT | AGCCAAGTTTCCTCGTGCC |
| *TRINITY_DN18429_c2* | CAGGGGAAGTATCACGGGTC | GCTCCAACACGAAGTCTCTGTAGTA |
| *TRINITY_DN18450_c0* | CCTACAAGTGTTCCGTAAGAGTTTT | TGGTGCCACAATCTCAGCC |
| *TRINITY_DN18469_c0* | ATCGCATCTCCGCCTCTTT | GATAACGACGGGAGAAGCAAG |
| *TRINITY_DN19069_c0* | GGGTTGCCATCAAATCTTACTG | CATCTTGGTTATGTTGCTCGG |
| *TRINITY_DN19221_c0* | TAGGGTTAGGGTGAGCACATAGA | TGTCTTGGGGATGTGTATGAATG |
| *TRINITY_DN22667_c2* | GGTGAAAATGGGCAACCTTAG | CCGTCCTTGCTCTTTGGTTC |
| *TRINITY_DN26458_c3* | CCCTGTTATTGTCTTCCTCCCA | AGCCGTATCTGTCGCTGTTG |
| *TRINITY_DN26898_c4* | GTTATGGTGTCGATTCAGTGGG | AACCTGCTTTCGCTCCCTG |
| *TRINITY_DN28256_c0* | GAGTGGGAACATCCAACGAAG | CGACAACACGAGGAATACGAGT |
| *TRINITY_DN28381_c6* | GCATCAGCACAAATCCCAAG | GGCGTTCCTATGACCACTTTC |
| *TRINITY_DN20551_c2-1* | TGTGGCTGATTACAATGGCG | GGTCGGCAATGTCCTTCCT |
| *TRINITY_DN20551_c2-2* | GAGCATCACGGCGAACAGT | AATGCTCAAGGCTGGTAATGC |

**Table S2.** Summary of identified metabolites

| Ion mode | All | Positive | Negative |
| --- | --- | --- | --- |
| the number of identified metabolites | 3,024 | 2,041 | 983 |

**Table S3.** Detected lignin-related metabolites.

| Index | Ion mode | Q1 (Da) | KEGG ID | Compounds | Class |
| --- | --- | --- | --- | --- | --- |
| pme0020 | Negative | 164.1 | C00079 | L-phenylalanine | amino acid and derivatives |
| pme0305 | Negative | 193.1 | C01494 | ferulic acid | phenylpropanoids |
| pme1436 | Negative | 163 | C00811 | p-coumaric acid | phenylpropanoids |
| pme1637 | Negative | 179.1 | C00590 | coniferyl alcohol | phenylpropanoids |
| pme2213 | Positive | 181 | C01197 | caffeate | phenylpropanoids |
| pme3123 | Negative | 209 | C02325 | sinapyl alcohol | phenylpropanoids |
| pme3305 | Negative | 149 | C02646 | p-coumaryl alcohol | phenylpropanoids |
| pme3443 | Negative | 207.1 | -- | sinapinaldehyde | phenylpropanoids |
| pme3453 | Negative | 147.1 | -- | p-Coumaraldehyde | phenylpropanoids |
| pmf0284 | Negative | 177.1 | C02666 | 4-hydroxy-3-methoxycinnamaldehyde | phenylpropanoids |

**Table S4.** Lignin-related DSMs.

| Group name | Total DSMs | Upregulated DSMs | Downregulated DSMs |
| --- | --- | --- | --- |
| Ss6_vs_Ss4 | 3 | 2 | 1 |
| Ss8_vs_Ss4 | 4 | 3 | 1 |
| Ss10_vs_Ss4 | 5 | 1 | 4 |
| Ss12_vs_Ss4 | 4 | 1 | 3 |
| Ss8_vs_Ss6 | 2 | 2 | 0 |
| Ss10_vs_Ss6 | 3 | 0 | 3 |
| Ss12_vs_Ss6 | 2 | 0 | 2 |
| Ss10_vs_Ss8 | 5 | 0 | 5 |
| Ss12_vs_Ss8 | 6 | 1 | 5 |
| Ss12_vs_Ss10 | 2 | 0 | 2 |

**Table S6.** Assembly result statistics.

| Item | Count |
| --- | --- |
| total trinity 'genes' | 147,798 |
| total trinity transcripts | 327,655 |
| median contig length | 499 |
| average contig | 925.18 |
| total assembled bases | 136,739,149 |
| contig N10 | 4,660 |
| contig N20 | 3,516 |
| contig N30 | 2,739 |
| contig N40 | 2,122 |
| contig N50 | 1,616 |

**Table S7.** Comparison result statistics.

| Sample id | Total reads | Total mapped reads | Unique match | Multi-position match | Percent of mapped reads (%) |
| --- | --- | --- | --- | --- | --- |
| Ss4_1 | 42,182,732 | 37,294,252 | 9,716,224 | 27,578,028 | 88.41 |
| Ss4_2 | 44,691,400 | 39,934,276 | 9,919,018 | 30,015,258 | 89.36 |
| Ss4_3 | 39,453,534 | 35,107,940 | 8,805,214 | 26,302,726 | 88.99 |
| Ss6_1 | 46,855,384 | 41,026,900 | 10,352,062 | 30,674,838 | 87.56 |
| Ss6_2 | 41,249,216 | 36,606,610 | 8,760,984 | 27,845,626 | 88.74 |
| Ss6_3 | 41,863,614 | 36,715,160 | 9,223,312 | 27,491,848 | 87.70 |
| Ss8_1 | 40,863,414 | 35,716,398 | 8,962,858 | 26,753,540 | 87.40 |
| Ss8_2 | 45,271,068 | 40,051,800 | 9,683,486 | 30,368,314 | 88.47 |
| Ss8_3 | 63,909,484 | 55,933,722 | 13,728,594 | 42,205,128 | 87.52 |
| Ss10_1 | 41,336,450 | 36,175,968 | 9,528,952 | 26,647,016 | 87.52 |
| Ss10_2 | 41,168,832 | 35,656,006 | 9,499,066 | 26,156,940 | 86.61 |
| Ss10_3 | 42,502,622 | 37,334,216 | 9,789,926 | 27,544,290 | 87.84 |
| Ss12_1 | 39,503,910 | 34,661,686 | 9,381,650 | 25,280,036 | 87.74 |
| Ss12_2 | 42,655,404 | 37,011,054 | 10,316,328 | 26,694,726 | 86.77 |
| Ss12_3 | 53,268,906 | 46,552,740 | 12,700,778 | 33,851,962 | 87.39 |

**Table S8.** Transcription factor prediction.

| TFs | Number | TFs | Number | TFs | Number |
| --- | --- | --- | --- | --- | --- |
| *ERF* | 36 | *Dof* | 8 | *ARR-B* | 2 |
| *bHLH* | 31 | *M-type_MADS* | 7 | *BES1* | 2 |
| *C2H2* | 29 | *SBP* | 7 | *GeBP* | 2 |
| *MYB* | 28 | *HD-ZIP* | 6 | *NF-X1* | 2 |
| *GRAS* | 24 | *MIKC_MADS* | 6 | *SAP* | 2 |
| *MYB_related* | 23 | *TCP* | 6 | *SRS* | 2 |
| *B3* | 21 | *DBB* | 5 | *AP2* | 1 |
| *FAR1* | 21 | *GATA* | 5 | *BBR-BPC* | 1 |
| *bZIP* | 18 | *GRF* | 5 | *CAMTA* | 1 |
| *WRKY* | 17 | *TALE* | 5 | *E2F/DP* | 1 |
| *C3H* | 16 | *ARF* | 4 | *NF-YA* | 1 |
| *NAC* | 14 | *NF-YB* | 4 | *RAV* | 1 |
| *Nin-like* | 13 | *CPP* | 3 | *Whirly* | 1 |
| *Trihelix* | 13 | *HB-other* | 3 | *WOX* | 1 |
| *G2-like* | 10 | *HSF* | 3 | *ZF-HD* | 1 |
| *LBD* | 10 | *NF-YC* | 3 |  |  |

**Table S11.** All samples difference analysis statistics.

| Sample id | Total | Up number | Down number |
| --- | --- | --- | --- |
| Ss6_vs_Ss4 | 2,738 | 1,307 | 1,431 |
| Ss8_vs_Ss4 | 5,247 | 2,921 | 2,326 |
| Ss10_vs_Ss4 | 8,946 | 4,322 | 4,624 |
| Ss12_vs_Ss4 | 12,522 | 6,222 | 6,300 |
| Ss8_vs_Ss6 | 4,631 | 2,608 | 2,023 |
| Ss10_vs_Ss6 | 9,910 | 4,944 | 4,966 |
| Ss12_vs_Ss6 | 13,813 | 6,918 | 6,895 |
| Ss10_vs_Ss8 | 5,078 | 2,270 | 2,808 |
| Ss12_vs_Ss8 | 12,196 | 5,754 | 6,442 |
| Ss12_vs_Ss10 | 9,676 | 4,419 | 5,257 |

**Table S12.** Differentially expressed transcription factors.

| TFs | Number | TFs | Number | TFs | Number |
| --- | --- | --- | --- | --- | --- |
| *ERF* | 23 | *G2-like* | 7 | *HSF* | 2 |
| *bHLH* | 18 | *Trihelix* | 6 | *SRS* | 2 |
| *C2H2* | 16 | *DBB* | 5 | *AP2* | 1 |
| *MYB* | 15 | *SBP* | 5 | *ARR-B* | 1 |
| *B3* | 12 | *TALE* | 5 | *BES1* | 1 |
| *bZIP* | 12 | *GATA* | 4 | *CAMTA* | 1 |
| *GRAS* | 12 | *GRF* | 4 | *E2F/DP* | 1 |
| *MYB_related* | 9 | *HD-ZIP* | 4 | *M-type_MADS* | 1 |
| *WRKY* | 9 | *LBD* | 4 | *NF-YA* | 1 |
| *C3H* | 8 | *TCP* | 4 | *NF-YC* | 1 |
| *NAC* | 8 | *ARF* | 3 | *RAV* | 1 |
| *Nin-like* | 8 | *CPP* | 3 | *WOX* | 1 |
| *Dof* | 7 | *MIKC_MADS* | 3 | *ZF-HD* | 1 |
| *FAR1* | 7 | *NF-YB* | 3 |  |  |
